# The male mosquito contribution towards malaria transmission: Mating influences the *Anopheles* female midgut transcriptome and increases female susceptibility to human malaria parasites

**DOI:** 10.1371/journal.ppat.1008063

**Published:** 2019-11-07

**Authors:** Farah Aida Dahalan, Thomas S. Churcher, Nikolai Windbichler, Mara K. N. Lawniczak

**Affiliations:** 1 Imperial College London, South Kensington, United Kingdom; 2 MRC Centre for Outbreak Analysis and Modelling, Department of Infectious Disease Epidemiology, Imperial College London, London, United Kingdom; 3 Wellcome Sanger Institute, Wellcome Genome Campus, Hinxton, United Kingdom; Institut Pasteur, FRANCE

## Abstract

Mating causes dramatic changes in female physiology, behaviour, and immunity in many insects, inducing oogenesis, oviposition, and refractoriness to further mating. Females from the *Anopheles gambiae* species complex typically mate only once in their lifetime during which they receive sperm and seminal fluid proteins as well as a mating plug that contains the steroid hormone 20-hydroxyecdysone. This hormone, which is also induced by blood-feeding, plays a major role in activating vitellogenesis for egg production. Here we show that female *Anopheles coluzzii* susceptibility to *Plasmodium falciparum* infection is significantly higher in mated females compared to virgins. We also find that mating status has a major impact on the midgut transcriptome, detectable only under sugar-fed conditions: once females have blood-fed, the transcriptional changes that are induced by mating are likely masked by the widespread effects of blood-feeding on gene expression. To determine whether increased susceptibility to parasites could be driven by the additional 20E that mated females receive from males, we mimicked mating by injecting virgin females with 20E, finding that these females are significantly more susceptible to human malaria parasites than virgin females injected with the control 20E carrier. Further RNAseq was carried out to examine whether the genes that change upon 20E injection in the midgut are similar to those that change upon mating. We find that 79 midgut-expressed genes are regulated in common by both mating and 20E, and 96% (n = 76) of these are regulated in the same direction (up vs down in 20E/mated). Together, these findings show that male *Anopheles* mosquitoes induce changes in the female midgut that can affect female susceptibility to *P*. *falciparum*. This implies that in nature, males might contribute to malaria transmission in previously unappreciated ways, and that vector control strategies that target males may have additional benefits towards reducing transmission.

## Author summary

Malaria mosquitoes must successfully mate and bloodfeed in order to reproduce. The impact of bloodfeeding on malaria transmission is clear given that all transmission is caused by female mosquitoes that have fed at least twice: once leading to an initial infection, and again 10–14 days later resulting in parasite transmission. The impact of mating on malaria transmission is less clear. Here we show that mating status significantly enhances transmission, such that mated females are more likely to transmit malaria parasites than virgin females. We further examine whether a hormone transferred by mating might cause this enhanced susceptibility, and we find that indeed the receipt of this hormone is also correlated with enhanced susceptibility. The results of this study imply that efforts to target male mosquitoes might not only suppress mosquito populations, but also act to decrease vector competence among residual females.

## Introduction

Malaria is a mosquito-transmitted parasitic disease, which still kills more than 400,000 people and infects more than 200 million people worldwide annually with 90% of the cases in Africa [1]. Malaria cases and mortality have shown dramatic reductions between 2000 and 2015, primarily due to vector control [2], but progress may be plateauing [1]. To date, the major strategies aimed at the vector are indoor residual spraying (IRS) of insecticides and long-lasting insecticide treated nets (LLINs) [3,4]. These control measures rely on continued mosquito susceptibility to various insecticides, however insecticide resistance has evolved to all four classes of insecticide currently in use [5] and new vector control strategies, including strategies that target reproduction, are sorely needed.

Several species of *Anopheles*, namely *An*. *gambiae*, *An*. *funestus*, and *An*. *coluzzii*, are responsible for the majority of transmission in Africa, where the disease causes the most morbidity [6]. *An*. *coluzzii*, formerly known as *An*. *gambiae* M form [7] is restricted to West and Central Africa compared to *An*. *gambiae* (formerly known as the S form) which is also found in the east and south. Like many insects, female *Anopheles* mosquitoes undergo behavioural changes after mating, including induction of egg laying and refractoriness to further insemination [8–12]. They also undergo major physiological changes: Transmission Electron Microscopy (TEM) experiments show mated female atrial cells are permanently altered as compared to virgin atrial cells in ways that suggest these cells function to support the uptake of male material transferred during copulation [10]. Comparisons of post mating gene expression between virgin and mated *An*. *gambiae* females suggests that mating causes permanent changes in gene expression [10], although immune genes have not been observed to be consistently regulated by mating as they are in *Drosophila* [10,13]. Differences between *An*. *gambiae* and *An*. *coluzzii* post-transcriptional responses in the lower reproductive tract and carcass have been observed, in particular with genes involved in reproduction and immunity [14]. For example, the important immune gene TEP1 is only found to be significantly upregulated in *An*. *gambiae* and not in *An*. *coluzzii*.

In *Aedes* and *Anopheles* mosquitoes, the titer of the hormone 20-hydroxyecdysone (20E) in females can be influenced by two major factors: blood-feeding and mating [15–17]. 20E is synthesized by the ovaries after a bloodmeal, and these levels are highest at 18 and 24 hours post blood feed [17]. Blood-feeding stimulates the ovary to secrete ecdysone, which is then hydroxylated to 20E by the fat body where it binds to the ecdysone receptor (EcR), which forms a dimer with ultraspiracle (USP) thereby activating the transcription of yolk protein precursors (YPP) such as vitellogenin (Vg) and lipophorin (Lp) [15,18,19]. Both Vg and Lp are lipid transporters important for egg production, and Vg is also the precursor protein of egg yolk. Thus, 20E derived from blood-feeding is involved in stimulating egg production. In addition to blood-feeding, mating also increases the titre of 20E. This is not due to endogenous production of 20E post-mating but rather due to females receiving a large dose of the hormone from the male. In *An*. *gambiae*, the male accessory gland (MAG) produces a high titre of 20E that gets incorporated into the mating plug, which is transferred to a female during mating [17]. The 20E derived from males interacts with a female protein known as Mating-Induced Stimulator of Oogenesis (MISO) in the atrium to regulate oogenesis and influence lipid accumulation in oocytes [20]. Apart from oogenesis, 20E also has a function in preserving the sperm in spermatheca by inducing heme peroxidase (HPX15) to ensure that sperm remain functional during storage given females typically only mate once in their life [21].

A microarray analysis of the atrium and spermatheca of *An*. *gambiae* females 24 hours post-injection into the thorax with 20E showed changes in gene expression patterns in the atrium and spermatheca that are very similar to gene expression patterns seen in these tissues 24 hours post-mating [12]. Indeed, both the induction of egg laying and refractoriness to further mating are male-induced and primarily driven by 20E and not by the sperm [12,22]. There is variability in the mating plug phenotypes of *Anopheles* species across the world. Ancestral reconstruction using maximum parsimony of mating plug phenotype and 20E titre shows a strong correlation between the solid and fully coagulated plug and a high titre of 20E produced in the MAG in four major malaria vector species *An*. *gambiae*, *An*. *arabiensis*, *An*. *stephensi*, and *An*. *funestus*, which suggests that 20E in particular might play a critical role in malaria transmission [23]. However, recent work calls this into question [24]. For example, *An*. *quadriannulatus* males produce the same level of 20E titre as *An*. *gambiae*, but this species is not a malaria vector. Furthermore, *An*. *quadrimaculatus*, *An*. *atroparvus*, *An*. *freeborni and An*. *albimanus* were highly susceptible to *P*. *falciparum* although 20E is not transferred by males.

The midgut of female *Anopheles* is a critical tissue for *P*. *falciparum* transmission, as parasites must mate in the bloodmeal and then migrate through the epithelial cells of the midgut to begin their vegetative growth and replication as oocysts. Here, we investigate the impact of both mating and 20E on female mosquito susceptibility to *P*. *falciparum*. We also use RNAseq to examine the transcriptomes of *An*. *coluzzii* female midguts that vary in their exposure to 20E, either due to bloodmeals, mating, or 20E injection.

## Results

### Infection susceptibility increases upon mating

**Fig 1:**
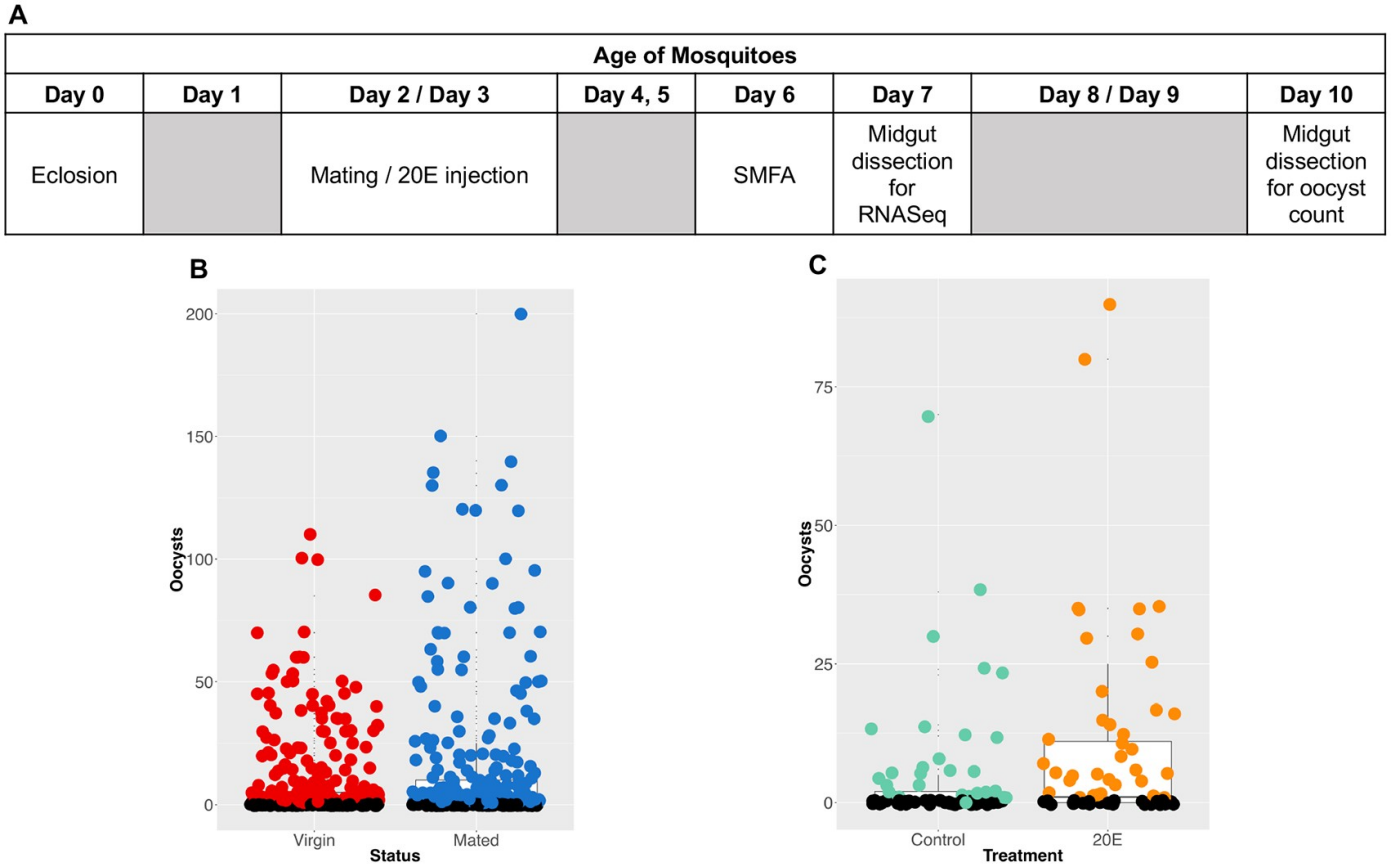
Mating and 20E injection both significantly enhance female susceptibility to *Plasmodium falciparum* infection. (A) Experimental design schematic for mating, 20E, bloodfeeds (i.e., SMFAs or Standard Membrane Feeding Assays), and RNASeq experiments. (B) Each dot is the number of *P*. *falciparum* oocysts counted in a single midgut dissected on day 10 post blood-feed. Data represented here are from 10 independent feeds (individually displayed in S1 Fig). Black dots are uninfected midguts (oocysts = 0). Boxplots indicate the median and 25-75th percentiles. Mated females show significantly higher infection intensity (*p* = 0.005) and prevalence (*p* = 0.039) compared to virgin females. (C) 20E injected females show significantly higher infection intensity (*p* < 0.001) and prevalence (*p* = 0.01) compared to control injected females.

To understand if mating status has an impact on female mosquito susceptibility to *Plasmodium falciparum*, we carried out standard membrane feeding assays (SMFAs) on virgin and mated *An*. *coluzzii* females using lab cultured NF54 *P*. *falciparum* gametocytes. The general experimental design followed the same timeline, with mating (or 20E injection) occurring 2 or 3 days post eclosion, and infectious blood feeding happening on day 6, followed by RNAseq on Day 7 or dissection 8–10 days post infection (Fig 1A), Across ten independent experiments with infection prevalence of 30% or more, we examined the impact of mating status on mosquito susceptibility to parasites using a generalised linear mixed-effect model. The best fit model indicated that oocyst intensity in females is significantly influenced by mating status **(***p* = 0.005, Fig 1B), with mated females on average having 38% higher oocyst loads than virgins (an average of 10.5 oocysts per mated midgut versus 7.6 oocysts in virgins). Similarly, prevalence is significantly affected by mating (*p* = 0.035), with mated females being 14% more likely to become infected (oocyst prevalence of 67% for mated females and 59% for virgins; Fig 1B). When looking at the individual feed level, while only 6 individual feeds out of 10 show that mated females have significantly higher infection intensity than virgin females, the higher infection intensity is, the more likely we are to see a significant increase in infection intensity in mated females (S1A Fig).

### 20E injection increases infection susceptibility in virgins

Our finding that mated females are more susceptible to *P*. *falciparum* than virgin females suggests that males potentially transfer substances during mating that can influence female susceptibility. We next tested whether the hormone 20E, which is known to be transferred in large quantities as part of the mating plug to the female during mating [17] might influence the outcome of infection. Virgin females were injected with a quantity of 20E previously determined to mimic mating [17] and compared to virgin controls that were injected with the carrier solvent alone (10% ethanol). Across three independent experiments, we find that 20E injected virgin females have significantly higher infection intensity compared to controls (*p* < 0.001, Fig 1C, S1B Fig), with an average of 2.7 oocysts in control injected virgins versus 8.5 oocysts in 20E injected virgins. Likewise, prevalence is also significantly affected by 20E injection (*p* < 0.001), with 20E injected females showing a 93% increase in prevalence (31% of control-injected virgins become infected, versus 60% of 20E injected virgins).

**Fig 2:**
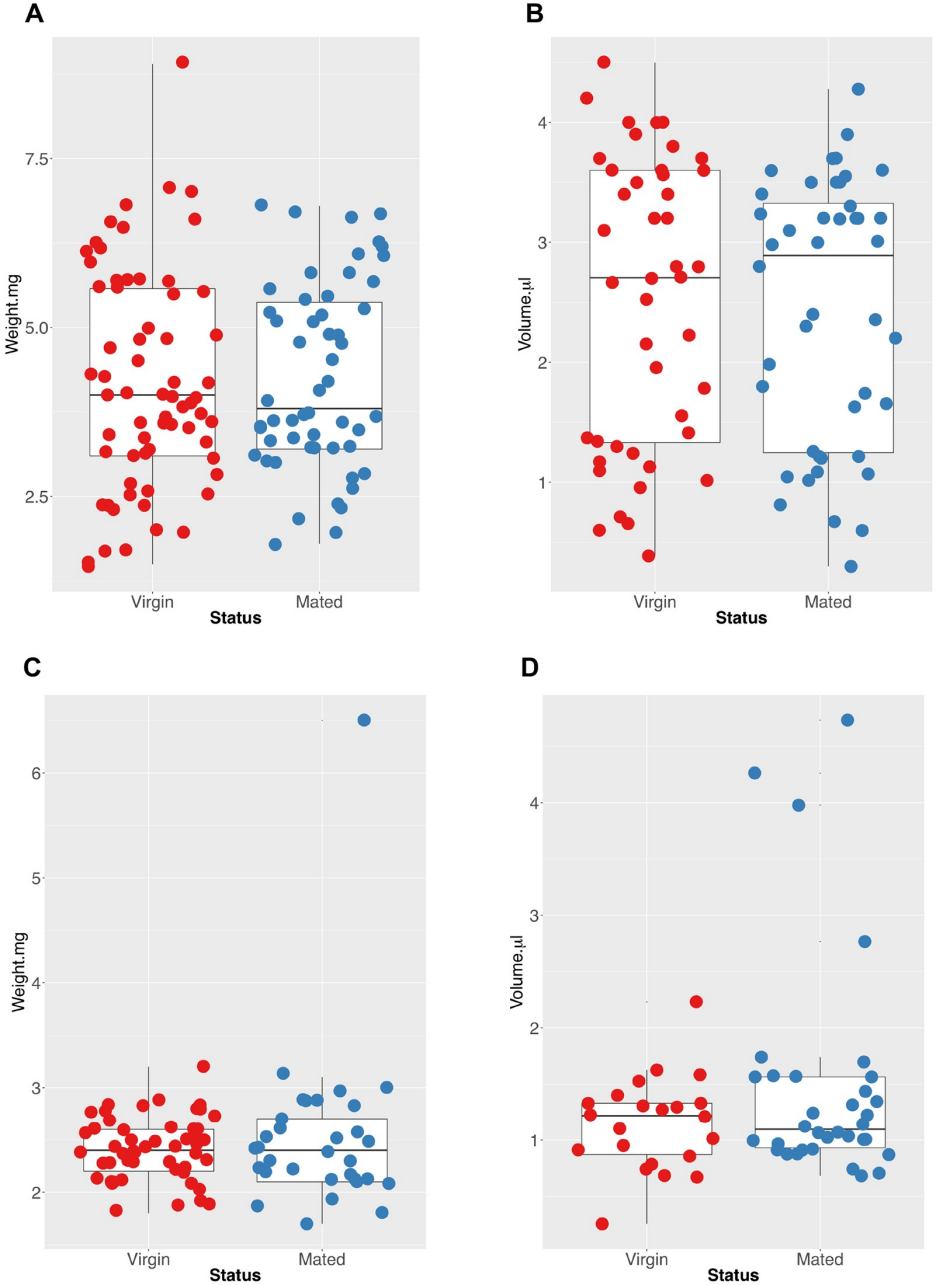
Mated and virgin females take similar bloodmeal volumes and digest to the same level after 24 hours. Boxplots indicate the median and 25-75th percentiles. Examining the (A) weights of blood-fed mosquitoes as well as their (B) hemoglobin levels using the Drabkin reagent shows blood-fed virgin and mated females take in the same the volume of blood at the time of feeding (0 hours post blood-meal). Furthermore, they digest to a similar level. Boxplots indicate the median and 25-75th percentiles. Examining the (C) weights of blood-fed mosquitoes immediately after a bloodmeal as well as their (D) hemoglobin levels using the Drabkin reagent reveals blood-fed virgin and mated females have digested blood to the same level as each other 24 hours post blood-meal. Boxplots indicate the median and 25-75th percentiles.

Differences between mated and virgin females in their susceptibility to parasites could be driven by different intake volumes and thus different parasite numbers rather than different susceptibility. Indeed there is evidence in *Drosophila* that females eat more after mating [25]. Different digestion rates could also influence infection outcomes as parasites do not invade the midgut for nearly a day. In order to examine whether the increase of infection in mated females was driven by differences in either the bloodmeal size between virgin and mated females, we tested whether they took different volumes of blood using two different measures: total weight and estimation of hemoglobin intake using the Drabkin method. The Drabkin method is a hemoglobin-based estimation of the blood meal taken by each mosquito [26]. Results from weighing and the Drabkin method are highly correlated (Correlation Test: R^2^ = 0.67), suggesting weight accurately represents bloodmeal size, and either method can be used to quantify the blood meal size taken in the future. Across three independent replicates for weight, and two for hemoglobin, we found that immediately after mating, virgin and mated females had indistinguishable weights (*p* = 0.6; Fig 2A) and hemoglobin content (*p* = 0.6; Fig 2B), and that 24 hours later, they also appeared to have digested blood to an indistinguishable endpoint by both weight (*p* = 0.4; Fig 2C) and hemoglobin content (*p* = 0.14; Fig 2D). This suggests that any differences detected between virgin and mated females in their infection rates are not driven by bloodmeal volume or digestion rate differences due to their mating status.

### The midgut transcriptome dramatically changes upon mating in sugar-fed females

We next explored the transcriptional patterns in the midguts of virgin and mated females that had fed on either sugar alone or on human parasite-infected blood. For experiments described below, we carried out RNAseq on individual female midguts (n = 6 replicate midguts for every treatment) with the exception of sugar-fed females. For these samples, it was challenging to get sufficient quantities of RNA from a single midgut to generate an RNA-sequencing library. Therefore, we pooled 7 dissected midguts for each of the six pools of virgin sugar-fed midguts, and 6 pools of mated sugar-fed midguts. These midguts were collected from experiment H.

**Fig 3:**
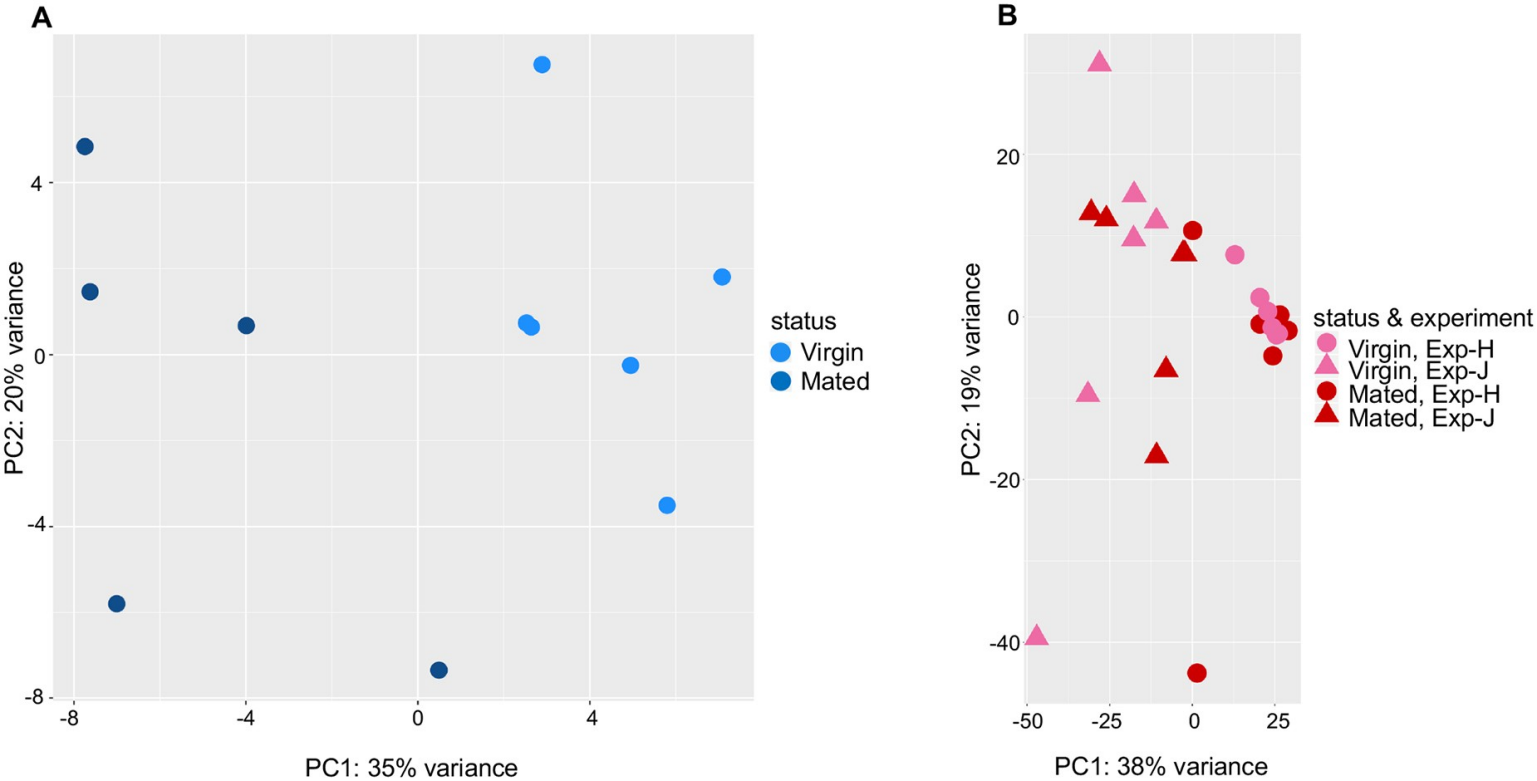
**Principal Components Analysis on RNAseq patterns** for (A) mated and virgin female midguts fed only on sugar. These midguts are from experiment H in S1 Fig. (B) PCA on global RNAseq patterns for mated and virgin female midguts fed on *P*. *falciparum* infective blood. The sugar-fed and *P*. *falciparum*-infected blood fed midguts are from Experiment H with 80% prevalence (circle) after *P*. *falciparum* infection whereas the other midguts are from experiment J, which achieved 100% prevalence (triangle).

In the sugar-fed midgut samples, one pool (P_M5) of mated females was a clear outlier (S2 Fig) and this sample was removed from downstream analysis to determine differentially expressed genes upon mating in the midgut. We find a clear impact of mating on sugar-fed midgut transcriptomes (Fig 3A). Using DESeq2 [27], we detected 479 genes to be significantly differentially expressed (*padj* <0.05) between virgin and mated female sugar-fed midguts. Among these, 311 genes were upregulated and 168 genes were downregulated upon mating (S1 Table), with upregulated genes showing the significant functional enrichment for gene ontologies involving sugar transport and the innate immune response, and down regulated genes in the mated midgut being enriched for translation (S1 File).

### The impact of mating on the midgut transcriptome is masked upon blood feeding

We next looked at the impact of mating on the midgut transcriptomes of females fed on *P*. *falciparum* infected bloodmeals. These females are from the same experimental replicate (experiment H) as the sugar-fed females, reared in the same way, and housed together until the time of infectious blood-feeding (three days post-mating). The single midguts examined using RNA-seq were harvested at 24 hours post-feed. The experiment from which we selected samples to use for RNAseq resulted in an 80% *P*. *falciparum* infection prevalence overall, so although we are unable to know for certain which guts would have become infected given we sample them 24 hours post-feed, approximately 80% of these individuals would have become infected. In contrast to the sugar-fed guts from the same experiment, only 6 genes were found to be significantly differentially expressed between virgin and mated blood-fed midguts (S1 Table). We also carried out RNAseq on an independent set of 6 virgin and 6 mated single midguts from an experiment (J) that had 100% *P*. *falciparum* infection prevalence. From these samples, we detected 33 genes significantly differentially expressed (30 upregulated upon mating, and 3 down regulated) between mated and virgin female midguts (S1 Table). From the Principal Component Analysis (PCA), the clear distinction of mateds from virgins that was observed among sugar-fed midguts is no longer seen for either replicate H or J (Fig 3B).

We also carried out a multifactor analysis to analyze all midgut samples, including sugar-fed and blood-fed from both experiments (experiment [H] and [J]). We find 7 genes significantly up-regulated and 7 genes significantly down-regulated upon mating (Table 1). One of the most significant of these, AGAP005656, was also detected as differentially expressed in previous studies examining mating [10] and the impact of 20E on the Lower Reproductive Tract (LRT) [12]. It is a cytochrome P450, and might be involved in oxidoreductase activity. The other top two genes, AGAP006508 and AGAP003523, also have no known function but are also predicted to be involved in ion binding and the oxidative stress response.

**Table 1:** List of genes differentially expressed in response to mating independent of feeding status. (*) represents 1-to-1 orthologue.

Gene ID	*p*-value	Upregulated	Putative Function	Drosophila Gene ID
AGAP005656	0.01	Mated	Oxidoreductase activity	FBgn0036910
AGAP006508	0.01	Mated	Transmembrane transport	FBgn0052103*
AGAP003523	0.01	Mated	Oxidoreductase activity	FBgn0264785*
AGAP005242	0.02	Mated	Unknown	FBgn0034021*
AGAP001874	0.02	Virgin	GTPase activity	FBgn0004636*
AGAP005498	0.02	Virgin	Phospholipid scramblase activity	FBgn0052056
AGAP008931	0.02	Virgin	Transmembrane transport	FBgn0010651
AGAP010682	0.02	Virgin	Gamma-glutamylaminecyclotransferase activity	FBgn0035082*
AGAP007801	0.02	Mated	DNA-binding transcription factor activity	FBgn0016076*
AGAP002114	0.03	Virgin	Positive regulation of ubiquitin protein ligase activity	FBgn0262699*
AGAP000693	0.05	Mated	Cecropin anti-microbial peptide	No orthologue
AGAP005163	0.05	Virgin	UDP-glycosyltransferase activity	FBgn0036842*
AGAP012018	0.05	Virgin	Neuron projection development	FBgn0037092*
AGAP012238	0.05	Mated	Phosphatase activity	No orthologue

### 20E injection changes the midgut transcriptome of female *An*. *coluzzii*

**Fig 4:**
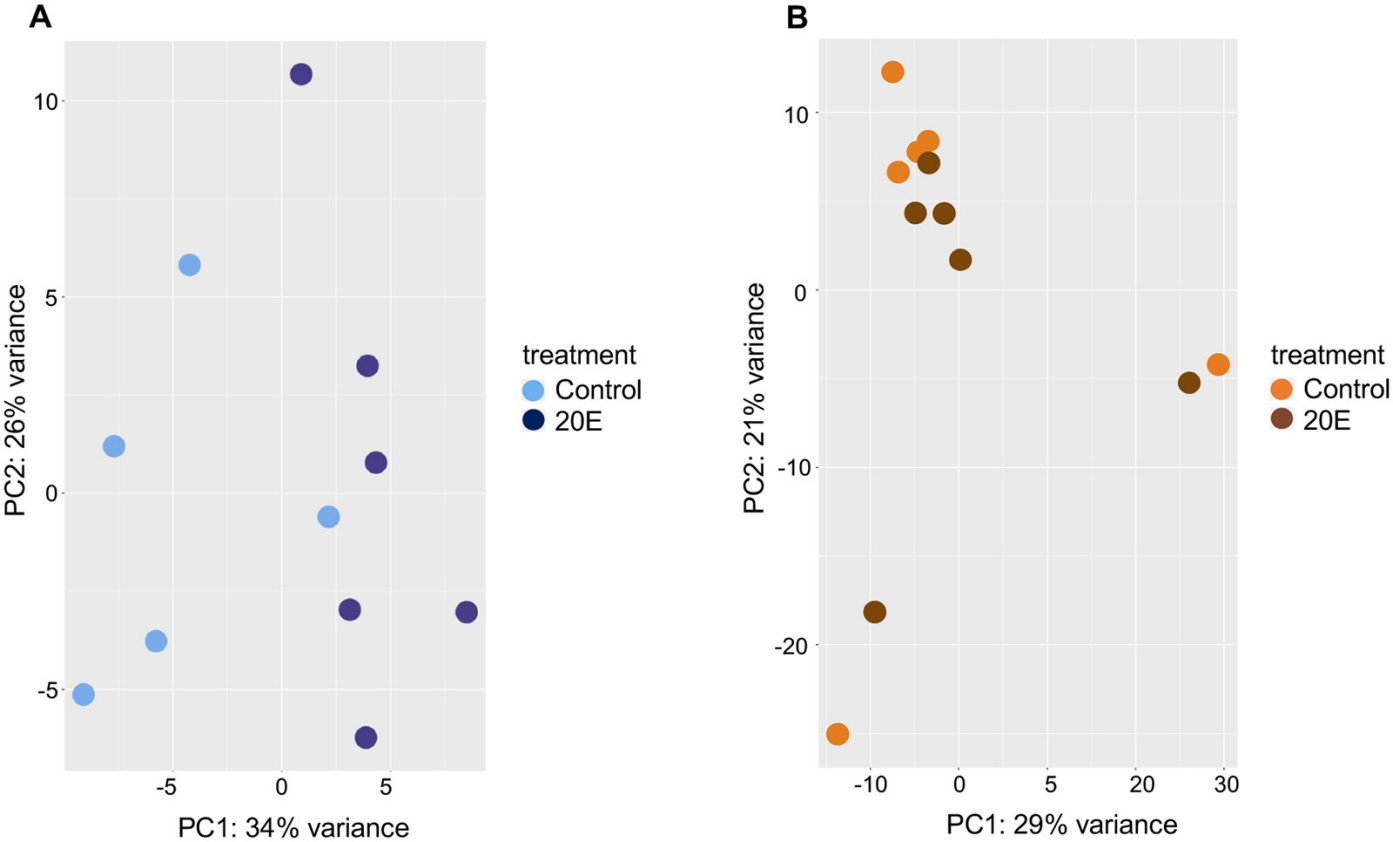
Principal Components Analysis on RNAseq data from midguts from (A) sugar-fed virgin females injected with 20E vs controls with each dot comprising data from a pool of 7 midguts, and (B) on single midguts from virgin females that were injected with 20E vs control and blood-fed on *P*. *falciparum* infective blood. All of these samples came from experiment [H] that achieved 80% prevalence after *P*. *falciparum* infection.

To understand if 20E injection has an impact on the midgut transcriptome, we examined the transcriptomes of both sugar-fed and blood-fed virgin midguts under different exposures to 20E. Similar to the difference observed between virgin and mated sugar-fed females, hundreds of genes are differentially expressed in 20E injected virgin midguts (n = six pools of 7 midguts each) vs control injected virgin midguts (n = six pools of 7 midguts each) and the transcriptomes are separated on the PCA (Fig 4A). We find 445 genes upregulated upon 20E injection and 731 genes downregulated compared to the control injected virgin pools. In general, the biological processes that were most significantly enriched among genes upregulated by 20E injection involve proton transport, whereas functional enrichment among genes downregulated upon 20E injection includes mainly protein phosphorylation (S1 File). Similar to blood-fed virgin versus mated midguts, virgins injected with 20E and then blood-fed show very few (n = 8) genes differentially expressed compared to virgins injected with 10% ethanol control (Fig 4B; S1 Table).

### The impact of 20E injection compared to mating on the midgut transcriptome

**Fig 5:**
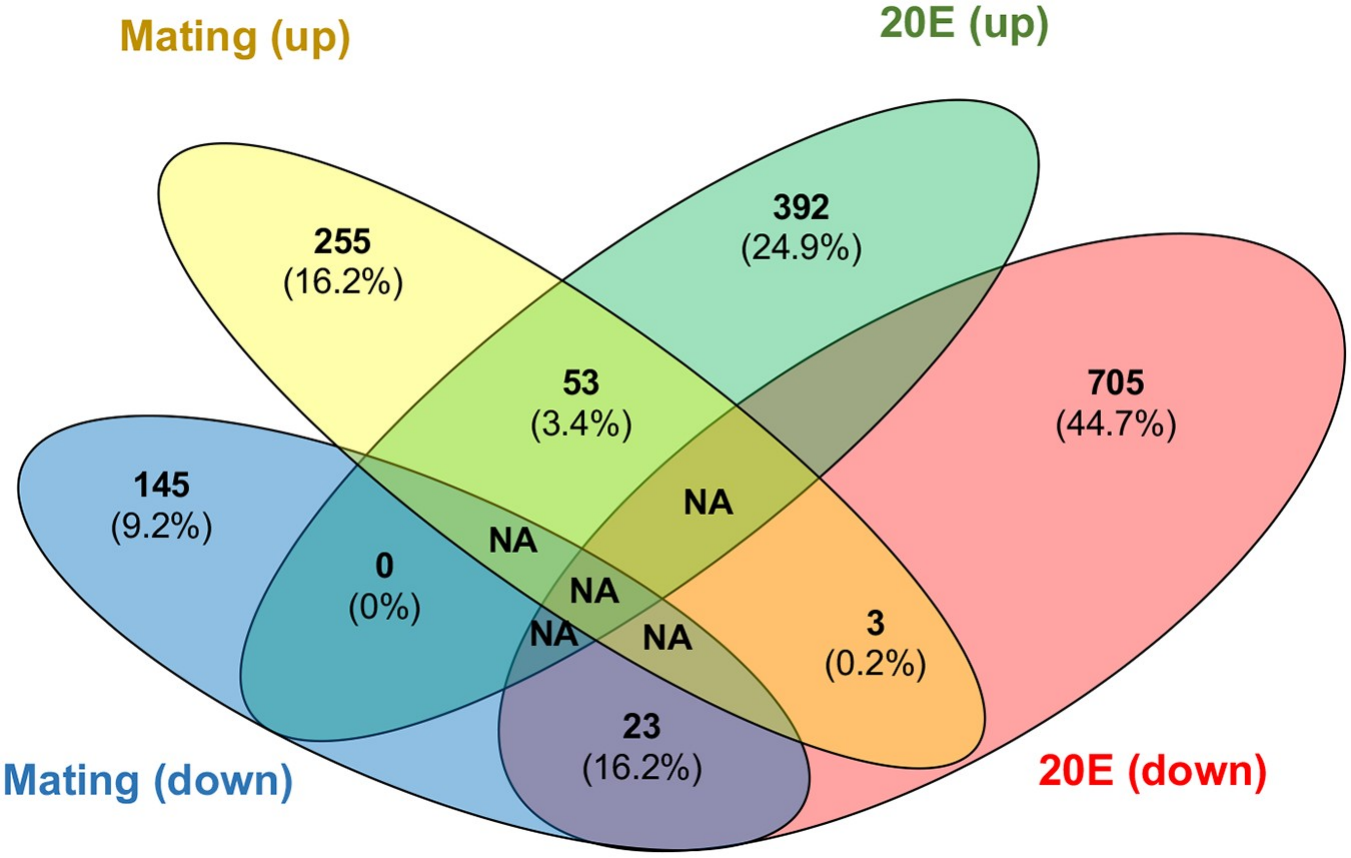
Venn diagram on number of genes that were upregulated and downregulated upon mating and 20E injection in sugar-fed virgin midguts. These samples were pools of 7 midguts per replicate and 5 to 6 replicates per condition. The midguts were dissected from the same batch (experiment H).

Above, we showed that both mating and 20E injection into virgin females resulted in a significant increase in *P*. *falciparum* infection intensity. This finding led us to hypothesize that 20E injection could result in transforming the virgin midgut into a mated female midgut, thus enhancing susceptibility to parasites. To further explore this at a gene level for sugar-fed females, we compared the overlap among genes significantly differentially expressed in the 20E-injection experiment (n = 1176 total) with those in the mating experiment (n = 479 total). We find that while only 79 genes are significantly influenced by both 20E and mating, of these all but 3 were influenced in the same direction: 56 genes were upregulated in mated females and 53 of these are also upregulated in 20E injected virgin midguts; 23 genes were downregulated in mated females and all of these were also downregulated in 20E injected virgin midguts (Fig 5; S1 Table). Analysis of the genes differentially expressed in sugar-fed female midguts by both mating and 20E injection shows some upregulated genes are enriched for functions in peptide catabolism, consistent with an enhanced ability to gain more nutrients from a bloodmeal. The genes affected are primarily metallopeptidases, and this may indicate shared function of 20E and mating to prepare the midgut to digest iron from the blood efficiently for egg provisioning [28]. Functional enrichment analysis for genes downregulated by both 20E injection and mating identifies two chymotrypsins putatively involved in digestion (S1 File).

While the directionality of expression changes for overlapping genes is nearly perfect, there are many genes that are not regulated in common by mating and 20E injection. Functional enrichment analysis for 400 genes significantly affected by mating but not 20E injection, and 1097 genes significantly affected by 20E injection but not by mating, tend to be involved in flavonoid biosynthesis or cytoskeleton reorganization, respectively (S1 File).

## Discussion

Our results support the idea that the mating status of an *Anopheles* female can significantly influence the outcome of her infection with the human malaria parasite *P*. *falciparum*. In the laboratory, we find that prior to a bloodmeal, mating dramatically influences the transcriptome of female midguts, and that mating predisposes a female to become more susceptible to *P*. *falciparum* parasites. In our lab colony, mosquitoes are kept under controlled conditions with constant access to food. This likely reduction of stress in the lab compared to the wild might enable mosquitoes to respond to infections differently than they would under natural conditions. It is possible that under more varied conditions or under more limited resource access at larval or adult stages, the difference between virgin and mated female susceptibility could be different, perhaps even enhanced further. We tested the hypothesis that male-derived 20E transferred during mating could cause the enhanced female susceptibility to infection that we observe among mated females by injecting virgins with 20E. Indeed, compared to control-injected females, females receiving a dose of 20E approximately equivalent to what the male transfers during mating are significantly more susceptible to human malaria parasites. Although injection is a different method of delivery of this hormone compared to mating, this result is suggestive that male-derived 20E may partially underlie enhanced female susceptibility to parasites.

In *Anopheles*, 20E is transferred to females during mating resulting in both induction of egg laying and refractoriness to further mating [12,22]. A previous study identified 628 genes that were regulated upon mating in the LRT and 459 of these genes were also found to be regulated by 20E injection in the LRT [12]. This suggests that much of the transcriptional change that occurs upon mating in the LRT may be driven by the receipt of 20E, consistent with 20E driving the phenotypic changes of induced egg laying and increased refractoriness to remating. We were curious to know if the midgut would reflect similar patterns whereby many of the transcriptional changes that are observed in the mated midgut are also observed in the midguts of 20E-injected virgins. We find that mating causes transcriptional changes of nearly 500 genes in the sugar-fed female midgut alone, whereas previous experiments investigating the impact of mating on the whole female transcriptome at 2, 6, and 24 hours post-mating revealed a total of 141 genes regulated by mating, with most genes differentially regulated at 24 hours post-mating [10]. Our RNAseq analyses were carried out 3 days post mating, and given the disparity in the number of genes influenced by mating (479 genes in the midgut in our study vs 141 genes in the whole body from [10]) might suggest that over time, the number of changes at the transcriptomic level due to mating increases, or that using the whole carcass (or microarrays) masks some of the changes that occur in specific tissues. In any case, it is clear that mating has a dramatic impact on midgut transcription in the female prior to a bloodmeal. Many of the functional categories that are overrepresented by genes that were upregulated reflect an impact of mating on sugar transport, metabolism, and the innate immune response (S1 File). In spite of blood feeding receiving the bulk of scientific attention, sugar feeding is also critical for female reproduction [29]. Females require sugar to search both for bloodmeals and for mates. Perhaps mated and virgin females use their sugar resources differently because virgin females must still find a mate even after a bloodmeal and might not provision eggs until they do so. In contrast, mated females no longer have to search for a mate, only for a blood meal. For example, mating in advance of a blood meal could prime the female midgut to direct resources towards egg provisioning rather than storing energy in the fat body as she no longer needs to use these resources to find a mate.

In contrast to the impact of mating on the sugar-fed midgut, blood-fed mated females (3 days post mating and 24 hours post bloodmeal) from two independent experiments show very few differentially expressed genes compared to blood-fed virgins. We explored the impact of mating on the midgut transcriptome of blood fed females from two independent experiments that had different *P*. *falciparum* infection prevalences (experiment [H] and [J] with 80% and 100% infection prevalence, respectively). These experiments showed only 6 and 33 significantly differentially expressed genes due to mating, respectively. It is possible that infection prevalence could influence the patterns we observed, however we suspect that the more likely explanation is that the massive transcriptional changes that occur to support blood digestion mask the difference between mated and virgin females, and that whatever consequence mating had on the midgut is no longer detectable by transcription after blood-feeding. Upon analysing all midgut samples (sugar-fed and blood-fed) in response to mating status, we found seven genes significantly upregulated upon mating. Most of these genes are predicted to be involved in oxidative stress response, transmembrane transport and one gene is an antimicrobial peptide which is active against bacteria and fungi.

Given that 20E injection has been shown to mimic the impact of mating in the LRT, with 459 of 628 mating-regulated genes also regulated by 20E [12,20], we next explored whether 20E injection of virgin females would mimic the impact of mating in the midgut. We find that the overlap of genes regulated by both 20E and mating is smaller than in the LRT (only 79 of 479 affected genes are in common), but the directionality is nearly perfect, with 53/56 upregulated genes in mated female midguts also showing upregulation in the 20E injection midguts, and 23/23 genes downregulated in mated females also downregulated in 20E injected midguts. The genes that significantly regulated only by mating and not by 20E (e.g., those not in common) showed enrichment of flavonoid biosynthesis and immune function, suggesting that mating status might influence the immune response in the midgut. Genes significantly regulated upon 20E injection but not by mating were mainly involved in cytoskeleton organization, suggesting 20E injection might be involved in midgut remodeling and consistent with what is observed in the female atrium [12]

In order to better understand potential function of differentially regulated genes, we examined what is known about their orthologs in *Drosophila*. In *Drosophila*, 20E produced by the ovary stimulates the central nervous system to accumulate nutrients used to provision eggs [30]. In *Drosophila*, mating induces the expression of Juvenile Hormone (JH) in females and this results in a remodeling of the midgut leading to cell division and ultimately a larger midgut [31]. This is partially accomplished by an increase in two basic Helix Loop Helix PAS domain proteins, Methoprene-tolerant (Met) and Germ cell-expressed (Gce) ultimately leading to increased female food absorption among mated females compared to virgins. Both of these *Drosophila* genes are orthologous to a single *Anopheles* gene, bhlh_PAS (AGAP006022), which is significantly downregulated by both mating and 20E injection in experiments presented here. *Drosophila* females have increased food intake initiated by compounds transferred during mating [32,33], but we did not detect a difference in the bloodmeal size or digestion rate of virgin compared to mated females. In *Drosophila*, this larger food intake among mated females is partially driven by mating increasing the size of the midgut, a phenomenon modulated by JH. The genes involved in lipid metabolism were increased upon mating which then increases egg production [31]. Although the impact of 20E injection on transcription in the *Anopheles* midgut does not fully mimic the impact of mating on midgut transcription, 20E injection is known to remodel the *Anopheles* female atrium [12]. This information, in combination with the work carried out in *Drosophila*, hints at the possibility that remodeling of the midgut might occur upon mating, preparing the midgut to divert resources to egg production. This possibility should be explored in future research. The mated midgut could also have more cells or larger cells than the virgin midgut potentially allowing more parasites to reside in the midgut and causing higher infection intensity. If the mated gut is remodeled to enhance nutrient absorption for egg development, the parasites could benefit from a reduced immune response resulting from most resources shifting towards egg provisioning. If true, this could also result in a higher parasite count. Mating and 20E injection has been reported to upregulate Lp expression [20]. Lp and Vg proteins are involved in delivering nutrients to nurture oocyte development, and have also been found to reduce TEP1 (thioester-containing protein) expression, thereby resulting in an increased infection with *P*. *berghei* [34]. We did not find these two genes to be differentially expressed in the midgut, but they may still influence outcomes of infection indirectly. In many insects, there is evidence to support the existence of tradeoffs between immunity and reproduction [35]. Female insects require energy for both of these activities. Many studies show female reproduction reduces immune responses towards infection [13,35] and other studies show that infection can reduce female reproductive capacity [35,36]. This tradeoff might be caused by limited resources needed by both immunity and reproduction, and is hypothesized to be regulated by the endocrine hormones, JH and 20E [35,37]. Recently, *P*. *falciparum* oocyst numbers were observed to be reduced when female-derived 20E was reduced [38]. In this paper, where underlying mechanisms explaining these results are also not discerned, the authors also hypothesise a trade-off scenario between immunity and reproduction [38].

Mating has been shown in other organisms to influence female gut physiology [31,39], however this has not been explored in *Anopheles*. It is particularly relevant for *Anopheles* and other blood feeding mosquitoes that transmit disease. If mating influences the outcome of infection by the pathogen/parasite, it is of medical relevance. Reducing mating opportunities by, for example, targeting males could be a potential control strategy that impacts not only fertilization rates but also malaria transmission. Previous work examining whole carcasses of mated females using microarrays shows that mating induced changes are long lasting [10]. This is expected as *An*. *gambiae* females mate only once and they may need to maintain transcriptional changes for successful reproduction. However, these previously observed differences are mainly in the lower reproductive tract of *An*. *gambiae*, and there are some differences even between the very closely related *An*. *gambiae* and *An*. *coluzzii* [14]. These differences will need further exploration to better understand if the results we present found here for *An*. *coluzzii* extend to *An*. *gambiae*.

Mimicking mating to disrupt the vector population could be an effective vector control strategy because of female monandry: if virgin females “think” they have been mated, this would effectively sterilize the population. Along these lines, a recent study found that the 20E agonist Dibenzoylhydrazine (DBH), which binds to the ecdysteroid receptor enhancing ecdysteroid activity, was able to reduce *P*. *falciparum* infection, prevent insemination, reduce egg laying, and reduce female lifespan [40]. This phenotype (reduced malaria) is the opposite to what was found here using 20E injection. We use *An*. *coluzzii* in our studies and it has been shown among wild collected of *An*. *coluzzii* and *An*. *gambiae* that upon mating, there are some differences in gene expression [14]. For example, only *An*. *coluzzii* upregulates MISO upon mating [14] and mating does not always increase egg production in *An*. *gambiae* [24]. Further exploration of the differences between *An*. *gambiae* and *An*. *coluzzii* are warranted as the recent study examining natural mosquitoes only explored a small subset of genes [14]. In any case, an agonist like DBH described above would have great benefits for malaria control and indeed, novel approaches that target vectors are sorely needed. However, based on the findings reported here, we would urge caution in targeting the 20E pathway given we find that increased 20E titers enhance transmission potential, opposite to the impact observed for DBH application. The relationship of 20E transfer by males during mating to malaria transmission is an area of active investigation, with some studies finding strong positive correlations between malaria transmission and the male-based transfer of 20E [23] and others finding no relationship [24]. If male-derived 20E enhances vector competence of mated females in nature, this suggests that males are contributing to malaria transmission in previously unappreciated ways. Therefore, proposed efforts to target male mosquitoes [41] might not only suppress mosquito populations, but also act to decrease vector competence among residual females.

## Materials and methods

### Mosquito rearing

The Ngousso strain of *An*. *coluzzii*, which originates from Cameroon, was reared under standard conditions (26°C-28°C, 65%-80% relative humidity, 12:12h light/darkness photoperiod). Eggs were floated in a pan filled with deionized water and once larvae hatched, around 1200 larvae were reared in a 32L plastic pan (66cm x 45cm x 17cm). The larvae were fed on 1 mL of ground fish food each day and 2 pieces of cat food on Friday evening for the weekend. When the adults emerged, they were maintained on 10% autoclaved fructose solution.

### Pupae sexing

Adult mosquitoes were separated by sex as pupae and placed in separate cages in dishes filled with deionized water. Cages were inspected when adults emerged and any of the wrong sexed adults were removed. After eclosion, “mated” females were aspirated into cages containing malesand “virgin” females were aspirated into a new cage lacking males. The aspirator was cleaned with 70% ethanol between uses.

### Mosquito mating

Mating assays were performed to determine the percentage of females mating over the course of one night. The spermathecae of females housed overnight with males were dissected the following morning and viewed under light microscope and 80% were found to have mated. Subsequently, we use a single overnight mating method in all experiments. Mated females and males were separated after they were left together overnight by putting the cage on ice. Virgin cages were treated the same way to account for any impact of cold exposure.

### Assessing bloodmeal volumes

In order to assess whether mated and virgin females take in different blood volumes, two experiments were performed, one which weighed females after their bloodmeal and another which used the Drabkin reagent to assess the amount of hemoglobin in each female’s gut (hemoglobinometry) [42]. Ngousso mosquitoes were fed using the membrane feeder on 1mL washed red blood cells (x3) mixed with 1mL A+ serum (Interstate Blood Bank). Serum was pooled from 4 different individuals. After 10 minutes of feeding, unfed females were removed on ice. Twenty females were separated and placed in a new cup on dry ice to kill them. They were then weighed individually and their mating status was scored by spermathecal dissection as described above. Remaining females were kept in a container in an incubator for 24 hours at 26°C and 80% humidity, similar to the condition with mosquitoes fed with *P*. *falciparum*-infected blood. These females were then weighed and mating status was assessed. This resulted in weights and Drabkin assessments of bloodmeal size for mated and virgin females both immediately after a bloodmeal, and 24 hours later to examine whether females may digest at different rates depending on their mating status. The Drabkin reagent (Sigma D5941) was prepared by mixing one vial of Drabkin reagent to 1L of water. Each mosquito abdomen was added to 0.5mL of Drabkin and mixed well, 200μL of mixture was pipetted into a 96-well plate and examined together with standard curve using normal blood; 0.5μL, 1.0μL, 2.0μL and 4μL. Controls of unfed mosquito abdomens with Drabkin and also Drabkin reagent alone were also included. Plates were read at 540nm and the standard curve was used to convert the OD value to blood volume in μL.

### 20E injection

20E injection was carried out to mimic mating in virgins [20] and to evaluate the impact on mated females. Virgin or mated females were injected in the thorax with either 2.5μg 20-hydroxyecdysone (H5142 Sigma) diluted in 10% ethanol at the thorax (20E injected females) or with 10% ethanol (control injected females). These injections were performed 2 or 3 days before blood feeding, early in the morning right after males were removed from the mated cage.

### *P*. *falciparum* gametocyte culturing

*P*. *falciparum* strain NF54 was cultured in complete RPMI 1640 (Invitrogen) supplemented with 10% human serum (mixed pooled serum from more than 4 individuals; Interstate Blood Bank), sodium bicarbonate (Sigma), hypoxanthine (Sigma) and D-glucose (Sigma). To induce *P*. *falciparum* gametocytes, cultures were set up to 0.75%–1% parasitemia at 6% hematocrit in complete RPMI 1640 medium. NHS Blood and Transplant (NHSBT) non-clinical use O+ blood was used after washing three times with incomplete medium (RPMI 1640 without serum). Culture medium was changed everyday and gassed for 15 seconds with 1% oxygen, 3% carbon dioxide and 96% nitrogen. *P*. *falciparum* stages on day 7 and day 14 was monitored under the microscope by Giemsa staining. In short, 3μL of *P*. *falciparum*-infected red blood cells were thin-smeared on a glass slide and fixed with methanol. Slides were stained with 20% Giemsa for 20 minutes, rinsed with water and dried before being assessed under 100x oil magnification light microscope.

An exflagellation assay was performed on day 14 and 17 to assess male gametocyte maturity. For these assays, 50μL of culture was placed on a glass slide and covered with a glass coverslip. Following 20 minutes of incubation at room temperature, the numbers of exflagellation centers were counted at magnification of 10x. Exflagellation is a process when the temperature drops and pH increases which induce the male gametes to undergo three times DNA replication and form eight highly motile flagellated microgametes. These microgametes will then fuse with female gametes to form zygotes in the vector’s gut. Only parasite cultures that showed exflagellation were used for feeds.

### Membrane feeding assay

Virgin and mated female Ngousso mosquitoes were prepared as described above. Sugar soaked cotton was removed six hours before blood feeding. Day 14/15 and 17/18 *P*. *falciparum* gametocyte cultures were pooled and spun down at 38°C, 2000rpm, for 5 minutes. Supernatant was removed and 1mL serum was added into the tube and topped up with fresh washed O+ blood to obtain a haematocrit of 45%. The mixture was always kept on a heat block (38°C) during the process to avoid inducing activation. The mixture was then added to a glass membrane feeder using a blunt syringe. Each cup of mosquitoes fed for 7 minutes and new sugar-soaked cotton was placed on each cup after feeding was completed. Cups were kept in a secure container in an incubator at 26°C and 80% humidity. Every unfed female was removed 24 hours after feeding. A sample of females had their midguts dissected (for RNAseq) and their mating status scored 24 hours post blood-feed, while all remaining fed females were retained in cups to assess infection rates at day 10 post blood-fed. Sugar-soaked cotton was changed every two days for all mosquitoes.

Oocyst counting occurred on day 10 post blood feed. Mosquitoes were killed using 70% ethanol followed by a 1X PBS wash. Midguts were dissected in 0.5% mercurochrome (Sigma) diluted in water with 1μL Hoescht added (1μg/ml) to assist with oocyst identification/counting. The guts were fixed in 4% Paraformaldehyde (PFA) for 30 minutes, then dipped in 1X PBS, and then left in another container of 1X PBS for 30 more minutes. Fixed and stained guts were mounted in Vectashield (Mounting Medium for fluorescence Vector Lab) and kept at 4°C until viewed. For each gut, the number of oocysts was counted under the microscope and recorded. Additionally, for each mated female, the spermatheca was dissected and if no sperm were observed, that midgut was discarded. Any virgin females identified from the mated cage were excluded because they might have been unhealthy or even potentially mated with no sperm stored.

### Statistical analyses

Differences in the oocyst intensity and prevalence between the individual experiment replicates were investigated using the Wilcoxon Exact Rank test and Chi Square test, respectively. Mosquito infection intensity and prevalence vary substantially between replicate experiments (i.e., mosquitoes fed at the same time on the same blood-source) and are difficult to control for. This variability was accounted for by including blood-source as a random effect within generalized linear mixed-effect models (package “glmmADMB” in R; http://glmmadmb.r-forge.r-project.org/) to generate an overall estimate of the impact of mating across all replicate experiments [43]. These models were used to investigate whether mating status in normal sugarfed females were predictors of infection intensity (i.e., number of oocysts). Similarly, we also evaluated the impact of 20E or control injection in virgin and mated females to test whether 20E influenced infection intensity. These different treatment types were included as fixed effects in a multivariate model (with all interaction terms). Nested versions of the full model were compared and the most parsimonious selected (and p-values generated) using a likelihood ratio test. Changes in oocyst prevalence were assessed assuming a binomial error structure. In all experiments only replicates which achieved at least 30% prevalence were included in the overall analysis.

### Molecular Biology

#### RNA extraction

Total RNA from female Ngousso midguts was extracted using TRIzol (Life Technologies) and chloroform (Sigma). For sugar-fed midguts, seven midguts were pooled together whilst for blood-fed midguts, each midgut was prepared individually. Each sample was homogenised using the Precellys 24 homogenizer at maximum speed, 6,800rpm for 30 seconds and let to rest for 5 minutes at room temperature (RT). Samples were then centrifuged at 13,200 rpm for 15 minutes at 4°C. The aqueous phase was collected into a tube that was filled with 250μL isopropanol (Sigma) and 1μg glycogen (Sigma) to precipitate the RNA. The pellet was kept to extract DNA using the back extraction buffer (described below). Samples were centrifuged at 13,200rpm for 15 minutes at 4°C followed by washing using 70% ethanol (Sigma) and concentrated using the DNA concentrator for 10 minutes. 10μL molecular biology grade water was added to each tube and was placed on a heat block (37°C) for 15 minutes. Total RNA was measured using Nanodrop.

#### RNASeq experiments

Subsets of mosquitoes as described above were kept aside specifically for RNA sequencing. At 24 hours post blood-fed, mosquito guts were dissected in RNAlater. Individual guts were placed in PCR tubes on dry ice as described above and RNA extraction was carried out as described above, on single or pooled guts depending on the experiment. These samples were assessed for RNA quality and quantity using the Agilent Bioanalyzer. Briefly, RNA samples were denatured by incubating at 70°C for 2 minutes and then placed on ice. 9μL of gel-dye mix was pipetted into bottom of nanochip. 1μL of samples, 1μL of RNA 6000 Nano Marker and 1μL of RNA 6000 Ladder were pipetted in assigned well, vortex and run using Eukaryote Total RNA Nano Series II programme. Total RNA was diluted to concentration of 500ng in 50μL and was sent to the Wellcome Sanger Institute for RNA library preparation and sequencing.

The kit used for library prep was Illumina TruSeq Stranded mRNA Library Prep Kit. mRNA was purified from total RNA using an oligo dT magnetic bead pull-down. A random-primed cDNA library was synthesized. During second strand synthesis dUTP was incorporated in place of dTTP. The incorporation of dUTP quenches the second strand during amplification because the polymerase does not incorporate past this nucleotide resulting in a strand specific library. Ends were repaired with a combination of fill-in reactions and exonuclease activity to produce blunt ends. A-tailing was performed, whereby an "A" base was added to the blunt ends. Illumina paired-end sequencing adapters containing unique index sequences, allowing samples to be pooled, were ligated. The libraries then went through 10 cycles of PCR amplification using KAPA Hifi Hot Start Polymerase rather than the kit-supplied Illumina PCR Polymerase due to better performance (especially with AT rich DNA). Libraries were quantified and pooled based on a post-PCR Agilent Bioanalyzer. Sequencing was done on the HiSeq 2500 v4, 75bp paired end reads, and the data was analysed using Illumina RTA software version 1.18.61. Automatic and manual quality control (QC) was performed and then the data was archived in iRODS as CRAM files. Data mapping to reference genome was done using TopHat2 (v.2.0.9) [44] which makes use of the aligner Bowtie2 (v.2.1.0) [45], to the *An*. *gambiae* PEST genome (AgamP4) obtained from VectorBase.

#### RNASeq analysis

Sequencing data received were run and mapped to reference genome of *An*. *gambiae* which was obtained from VectorBase. HTSeq (v.0.6.1) [46] was used to count transcripts for each gene. Differentially expressed genes in response to mating and/or 20E injection were determined using DESeq2 (v.1.8.2) [27,47] in R (v.3.2.5) (R Core Team 2014). Independent filtering was turned on to filter low count genes to increase sensitivity for the dataset as a whole to reduce false negative. The genes with adjusted p-value of NA have less mean normalized counts than the optimal threshold. To analyse the impact of mating on all of the samples, a multifactor analysis was undertaken, controlling for the impact of different sets of experiment, different types of feed (either *P*. *falciparum*-infected blood or sugar), and sample type (either single midgut or pooled guts) as additional factors. Principal Component Analysis was used in R to estimate sampling distribution in every experiment. Overlapping genes from RNASeq analysis was performed using Venn Diagram [47]. Functional interpretation of each gene set from DESeq2 was performed by doing Gene Ontology analysis using web server, TopGO [48,49].

## Supporting information

S1 Fig (A) Each dot represents number of oocysts per single midgut dissected on day 10 post blood feed. Boxplots indicate the median and 25–75 percentiles. *P*. *falciparum* infection intensity in virgin and mated female midguts from each individual experiment summarized in Fig 1A. Six individual feeds out of 10 show that mated females have significantly higher infection intensity than virgin females; Wilcoxon Rank Test: A (*p* = 0.09), **B (*p* = 0.05)**, **C (*p* = 0.04)**, **D (*p* = 0.05)**, E (*p* = 0.2), F (*p* = 0.85), **G (*p* = 0.0002)**, **H (*p* = 0.003)**, **I (*p* = 0.004)**, J (*p* = 0.06). (B) Overall *P*. *falciparum* infection intensity in virgin female midguts which were injected with 20E or control (10% EtOH) from three separate experiments summarized in Fig 1B. One of three individual experiments showed a significant impact of 20E injection increasing infection intensity Wilcoxon Rank Test: A: *p* = 0.19 **F: *p* = 0.003**, H: *p* = 0.07).
(TIF)

S2 Fig Principal Components Analysis on RNAseq patterns for mated and virgin female midguts fed only on sugar. “P_” indicates the sample is from a pooled of 7 midguts in a sample, M is mated, V is virgin and the numbers are the replicates. These midguts are from experiment H. P_M5 was identified as a major outlier and removed from further analyses.
(TIF)

S1 File Functional enrichment analysis for differentially expressed genes in the midgut. (DOCX)

S1 Table List of all annotated genes in *Anopheles gambiae* and how they are affected by mating in both sugarfed and bloodfed midguts as well as by 20E injection. (XLSX)
