# Supplementary figures and images for: The male mosquito contribution towards malaria transmission: Mating influences the Anopheles female midgut transcriptome and increases female susceptibility to human malaria parasites
Source: PLoS Pathog. 2019 Nov 7;15(11):e1008063. doi: 10.1371/journal.ppat.1008063 (PMC6837289; doi:10.1371/journal.ppat.1008063)

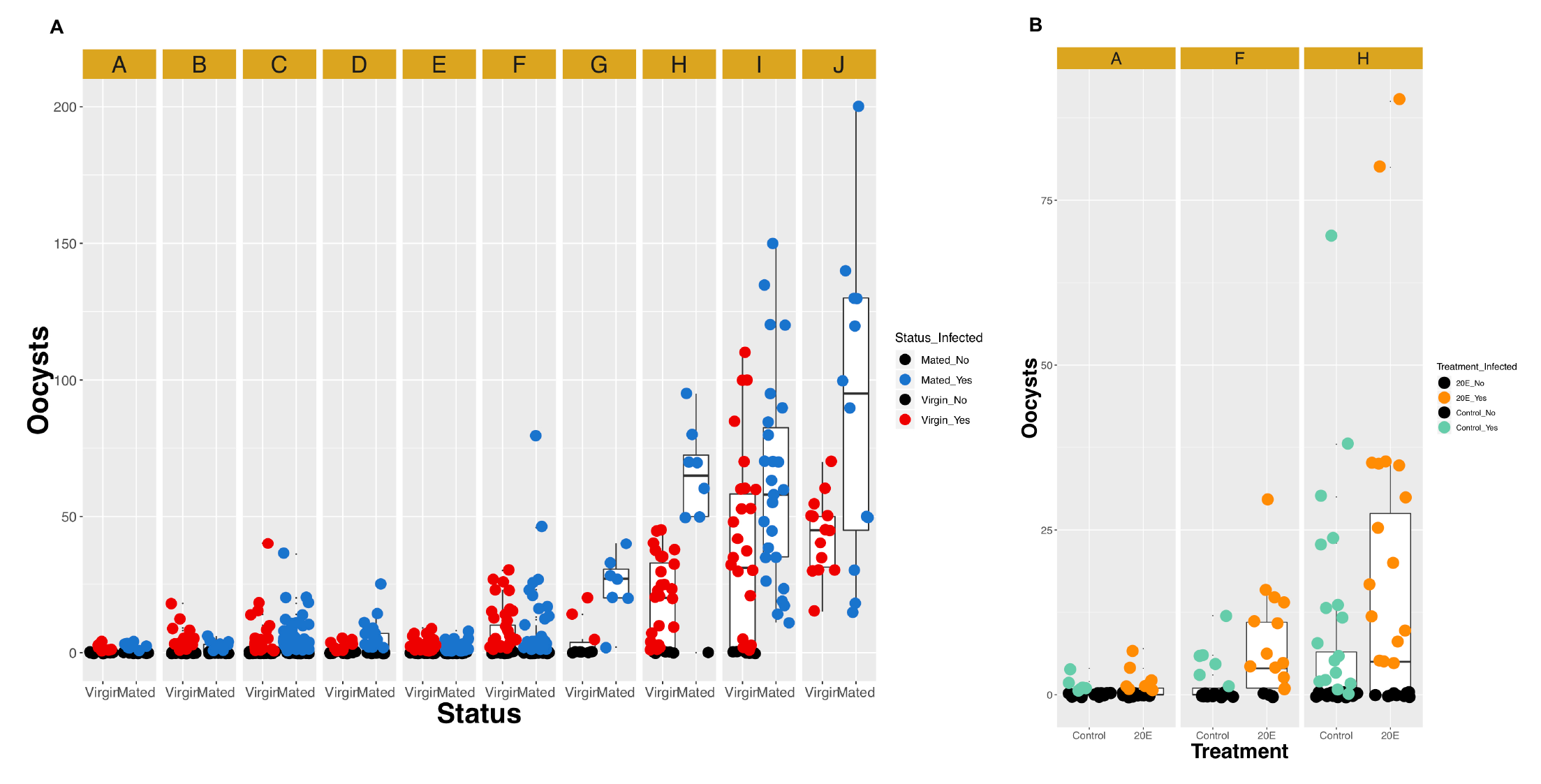

Supplement: S1 Fig — (A) Each dot represents number of oocysts per single midgut dissected on day 10 post blood feed. Boxplots indicate the median and 25–75 percentiles. P. falciparum infection intensity in virgin and mated female midguts from each individual experiment summarized in Fig 1A. Six individual feeds out of 10 show that mated females have significantly higher infection intensity than virgin females; Wilcoxon Rank Test: A (p = 0.09), B (p = 0.05), C (p = 0.04), D (p = 0.05), E (p = 0.2), F (p = 0.85), G (p = 0.0002), H (p = 0.003), I (p = 0.004), J (p = 0.06). (B) Overall P. falciparum infection intensity in virgin female midguts which were injected with 20E or control (10% EtOH) from three separate experiments summarized in Fig 1B. One of three individual experiments showed a significant impact of 20E injection increasing infection intensity Wilcoxon Rank Test: A: p = 0.19 F: p = 0.003, H: p = 0.07). (TIF) [file ppat.1008063.s001.tif]

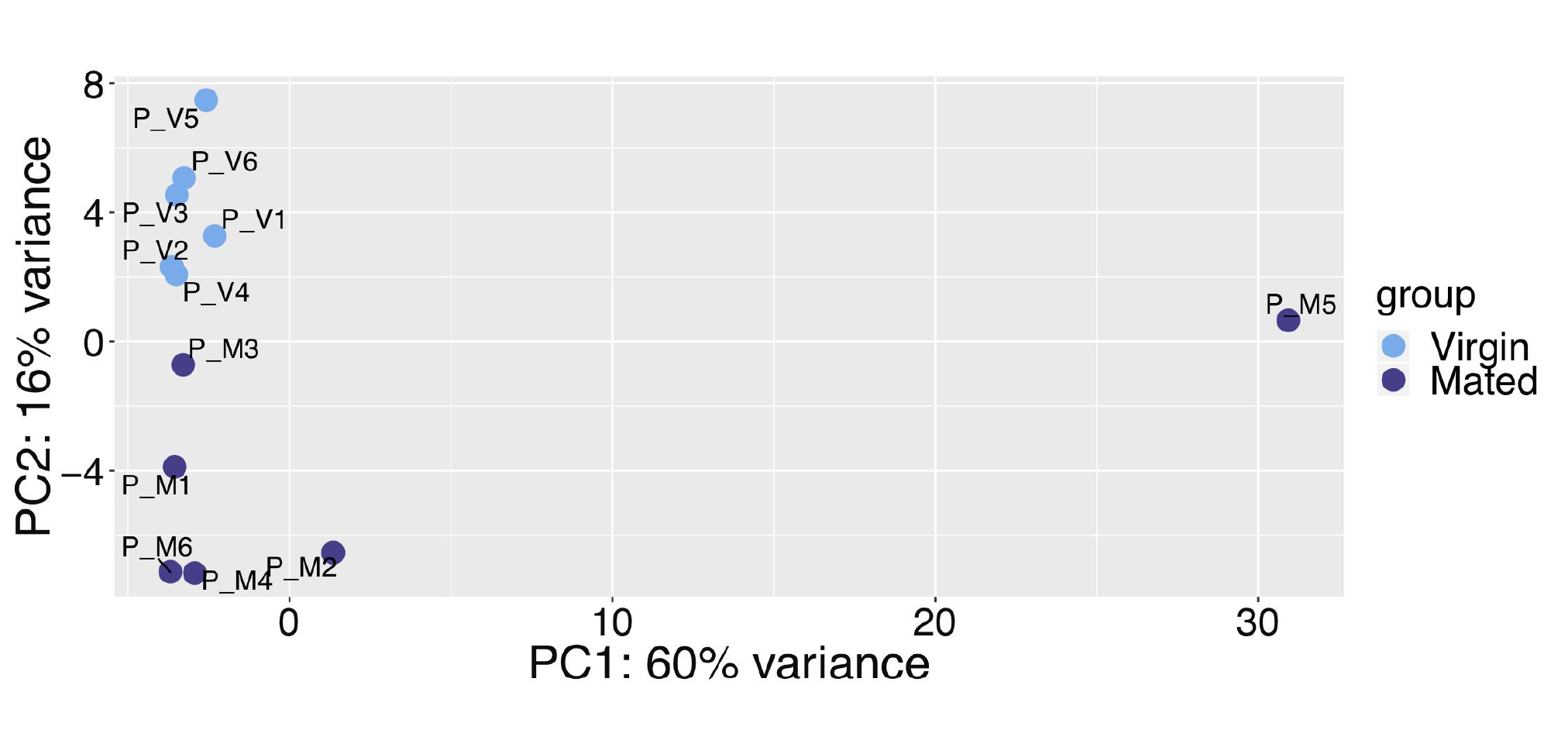

Supplement: S2 Fig — “P_” indicates the sample is from a pooled of 7 midguts in a sample, M is mated, V is virgin and the numbers are the replicates. These midguts are from experiment H. P_M5 was identified as a major outlier and removed from further analyses. (TIF) [file ppat.1008063.s002.tif]
