# Supplementary material for: The male mosquito contribution towards malaria transmission: Mating influences the Anopheles female midgut transcriptome and increases female susceptibility to human malaria parasites
Source: PLoS Pathog. 2019 Nov 7;15(11):e1008063. doi: 10.1371/journal.ppat.1008063 (PMC6837289; doi:10.1371/journal.ppat.1008063)
Supplement: S1 File — (DOCX) [file ppat.1008063.s003.docx]

**Gene Enrichment Analysis on list of genes that were significantly upregulated in sugar fed mated female midguts compared to sugar fed virgin female midguts**

**Biological Process**

GO.ID Term Annotated Significant Expected weight01

1 GO:0046323 glucose import 17 6 0.56 1.1e-05

2 GO:0009813 flavonoid biosynthetic process 19 6 0.63 2.3e-05

3 GO:0052696 flavonoid glucuronidation 19 6 0.63 2.3e-05

4 GO:0042742 defence response to bacterium 10 4 0.33 0.00021

5 GO:0045087 innate immune response 31 6 1.02 0.00044

6 GO:0055085 transmembrane transport 426 31 14.07 0.00110

7 GO:0043171 peptide catabolic process 29 5 0.96 0.00232

8 GO:0046701 insecticide catabolic process 21 4 0.69 0.00444

9 GO:0046680 response to DDT 21 4 0.69 0.00444

10 GO:0003333 amino acid transmembrane transport 23 4 0.76 0.00624

11 GO:0006749 glutathione metabolic process 37 5 1.22 0.00689

12 GO:0009074 aromatic amino acid family catabolic process 5 2 0.17 0.01016

13 GO:0032787 monocarboxylic acid metabolic process 108 10 3.57 0.01952

14 GO:0006767 water-soluble vitamin metabolic process 7 2 0.23 0.02042

15 GO:0006810 transport 1050 57 34.67 0.02688

16 GO:0046131 pyrimidine ribonucleoside metabolic process 9 2 0.30 0.03290

17 GO:0018208 peptidyl-proline modification 15 2 0.50 0.03293

18 GO:1901570 fatty acid derivative biosynthetic process 17 2 0.56 0.03294

19 GO:0009253 peptidoglycan catabolic process 9 2 0.30 0.03352

20 GO:0007586 digestion 10 2 0.33 0.04100

21 GO:0006541 glutamine metabolic process 10 2 0.33 0.04100

**Cellular Compartment**

GO.ID Term Annotated Significant Expected weight01

1 GO:0005887 integral component of plasma membrane 271 19 7.42 0.00024

2 GO:0043231 intracellular membrane-bounded organelle 2053 42 56.21 0.00158

3 GO:1902495 transmembrane transporter complex 46 3 1.26 0.00432

4 GO:0005791 rough endoplasmic reticulum 5 2 0.14 0.00706

5 GO:0005885 Arp2/3 protein complex 7 2 0.19 0.01429

6 GO:0016020 membrane 2818 99 77.16 0.02407

7 GO:0000139 Golgi membrane 48 4 1.31 0.04146

8 GO:0005737 cytoplasm 1724 55 47.20 0.04553

**Molecular Function**

GO.ID Term Annotated Significant Expected weight01

1 GO:0005355 glucose transmembrane transporter activity 17 6 0.44 2.8e-06

2 GO:0005351 carbohydrate:proton symporter activity 20 6 0.52 8.1e-06

3 GO:0015171 amino acid transmembrane transporter activity 31 5 0.80 0.0012

4 GO:0016671 oxidoreductase activity, acting on a sulphur 9 3 0.23 0.0013

5 GO:0016831 carboxy-lyase activity 25 5 0.65 0.0015

6 GO:0004602 glutathione peroxidase activity 21 4 0.55 0.0019

7 GO:0022857 transmembrane transporter activity 500 30 12.98 0.0033

8 GO:0070006 metalloaminopeptidase activity 26 4 0.67 0.0042

9 GO:0008194 UDP-glycosyltransferase activity 41 6 1.06 0.0047

10 GO:0004364 glutathione transferase activity 27 4 0.70 0.0048

11 GO:0003824 catalytic activity 3379 124 87.70 0.0052

12 GO:0004190 aspartic-type endopeptidase activity 6 2 0.16 0.0094

13 GO:0016853 isomerase activity 76 9 1.97 0.0204

14 GO:0008745 N-acetylmuramoyl-L-alanine amidase activity 9 2 0.23 0.0214

15 GO:0016709 oxidoreductase activity 10 2 0.26 0.0263

16 GO:0003756 protein disulfide isomerase activity 10 2 0.26 0.0263

17 GO:0016758 transferase activity 89 8 2.31 0.0283

18 GO:0042277 peptide binding 50 4 1.30 0.0401

19 GO:0004497 monooxygenase activity 110 8 2.86 0.0438

20 GO:0008028 monocarboxylic acid transmembrane transport 14 2 0.36 0.0497

21 GO:0015020 glucuronosyltransferase activity 14 2 0.36 0.0497

22 GO:0020037 heme binding 156 8 4.05 0.0500

**Gene Enrichment Analysis on list of genes that were significantly upregulated in sugar fed virgin female midguts compared to sugar fed mated female midguts**

**Biological Process**

GO.ID Term Annotated Significant Expected weight01

1 GO:0000027 ribosomal large subunit assembly 15 4 0.26 0.00010

2 GO:0000479 endonucleolytic cleavage of tricistronic rRNA 8 3 0.14 0.00027

3 GO:0000055 ribosomal large subunit export from nucleus 5 2 0.09 0.00293

4 GO:0031126 snoRNA 3'-end processing 6 2 0.10 0.00434

5 GO:0006414 translational elongation 20 3 0.35 0.00477

6 GO:0006783 heme biosynthetic process 7 2 0.12 0.00601

7 GO:0000154 rRNA modification 17 3 0.30 0.00780

8 GO:1901657 glycosyl compound metabolic process 49 3 0.86 0.00788

9 GO:0000463 maturation of LSU-rRNA 8 2 0.14 0.00792

10 GO:0009253 peptidoglycan catabolic process 9 2 0.16 0.01007

11 GO:0007586 digestion 10 2 0.17 0.01244

12 GO:0010501 RNA secondary structure unwinding 29 3 0.51 0.01366

13 GO:0000462 maturation of SSU-rRNA 30 3 0.52 0.01499

14 GO:0002181 cytoplasmic translation 35 3 0.61 0.02269

15 GO:0000466 maturation of 5.8S rRNA 14 2 0.24 0.02405

16 GO:0071428 rRNA-containing ribonucleoprotein complex 10 3 0.17 0.03408

17 GO:0009813 flavonoid biosynthetic process 19 2 0.33 0.04270

18 GO:0052696 flavonoid glucuronidation 19 2 0.33 0.04270

**Cellular Compartment**

GO.ID Term Annotated Significant Expected weight01

1 GO:0005730 nucleolus 87 11 1.35 0.00017

2 GO:0044452 nucleolar part 30 5 0.46 0.00025

3 GO:0022625 cytosolic large ribosomal subunit 43 5 0.67 0.00048

4 GO:0005732 small nucleolar ribonucleoprotein complex 12 3 0.19 0.00071

5 GO:0030688 preribosome, small subunit precursor 5 2 0.08 0.00230

6 GO:0032040 small-subunit processome 29 3 0.45 0.00980

7 GO:0005654 nucleoplasm 149 4 2.31 0.02472

8 GO:0030686 90S preribosome 22 2 0.34 0.04478

9 GO:0030687 preribosome, large subunit precursor 23 2 0.36 0.04856

**Molecular Function**

GO.ID Term Annotated Significant Expected weight01

1 GO:0003746 translation elongation factor activity 13 3 0.21 0.0010

2 GO:0008422 beta-glucosidase activity 6 2 0.10 0.0037

3 GO:0004659 prenyltransferase activity 8 2 0.13 0.0068

4 GO:0003735 structural constituent of ribosome 173 8 2.80 0.0068

5 GO:0008745 N-acetylmuramoyl-L-alanine amidase activity 9 2 0.15 0.0087

6 GO:0005548 phospholipid transporter activity 9 2 0.15 0.0087

7 GO:0030515 snoRNA binding 14 2 0.23 0.0208

8 GO:0015020 glucuronosyltransferase activity 14 2 0.23 0.0208

9 GO:0004004 ATP-dependent RNA helicase activity 38 3 0.61 0.0231

10 GO:0004521 endoribonuclease activity 18 2 0.29 0.0335

11 GO:0032549 ribonucleoside binding 145 5 2.34 0.0471

12 GO:0003723 RNA binding 332 16 5.37 0.0481

**Gene Enrichment Analysis on list of genes that were significantly upregulated in sugar fed 20E injected virgin female midguts compared to sugar fed control injected virgin female midguts**

**Biological Process**

GO.ID Term Annotated Significant Expected weight01

1 GO:0015991 ATP hydrolysis coupled proton transport 22 7 0.85 1.2e-05

2 GO:0006890 retrograde vesicle-mediated transport 11 5 0.42 3.1e-05

3 GO:0015986 ATP synthesis coupled proton transport 21 6 0.81 0.00010

4 GO:0051603 proteolysis involved in cellular protein 141 14 5.44 0.00013

5 GO:0034314 Arp2/3 complex-mediated actin nucleation 12 4 0.46 0.00084

6 GO:0006418 tRNA aminoacylation for protein translation 44 7 1.70 0.00131

7 GO:0045047 protein targeting to ER 12 5 0.46 0.00145

8 GO:0006465 signal peptide processing 7 3 0.27 0.00177

9 GO:0006891 intra-Golgi vesicle-mediated transport 16 4 0.62 0.00272

10 GO:0006122 mitochondrial electron transport 8 3 0.31 0.00275

11 GO:0015031 protein transport 223 21 8.60 0.00450

12 GO:0043171 peptide catabolic process 29 5 1.12 0.00455

**Cellular Compartment**

GO.ID Term Annotated Significant Expected weight01

1 GO:0033179 proton-transporting V-type ATPase, V0 domain 9 5 0.42 2.2e-05

2 GO:0005737 cytoplasm 1724 133 79.75 7.9e-05

3 GO:0005885 Arp2/3 protein complex 7 4 0.32 0.00014

4 GO:0019773 proteasome core complex, alpha-subunit 7 4 0.32 0.00014

5 GO:0005739 mitochondrion 353 43 16.33 0.00043

6 GO:0005789 endoplasmic reticulum membrane 97 14 4.49 0.00050

7 GO:0030126 COPI vesicle coat 6 3 0.28 0.00176

8 GO:0016471 vacuolar proton-transporting V-type ATPase 6 3 0.28 0.00176

9 GO:0000276 mitochondrial proton-transporting ATP synthase 7 3 0.32 0.00298

10 GO:1905368 peptidase complex 45 11 2.08 0.00431

11 GO:0005839 proteasome core complex 15 7 0.69 0.00444

12 GO:0098796 membrane protein complex 251 34 11.61 0.01511

13 GO:0005753 mitochondrial proton-transporting ATP synthase 12 5 0.56 0.01907

14 GO:0005791 rough endoplasmic reticulum 5 2 0.23 0.01943

15 GO:0005750 mitochondrial respiratory chain complex 5 2 0.23 0.01943

16 GO:0005747 mitochondrial respiratory chain complex 14 3 0.65 0.02439

17 GO:0005688 U6 snRNP 6 2 0.28 0.02826

18 GO:0031902 late endosome membrane 6 2 0.28 0.02826

19 GO:0033180 proton-transporting V-type ATPase, V1 domain 7 2 0.32 0.03838

20 GO:0048500 signal recognition particle 7 2 0.32 0.03838

21 GO:0030173 integral component of Golgi membrane 8 2 0.37 0.04963

22 GO:0005758 mitochondrial intermembrane space 8 2 0.37 0.04963

**Molecular Function**

GO.ID Term Annotated Significant Expected weight01

1 GO:0004298 threonine-type endopeptidase activity 15 7 0.48 1.6e-07

2 GO:0046961 proton-transporting ATPase activity 16 6 0.51 6.0e-06

3 GO:0051117 ATPase binding 5 3 0.16 0.00030

4 GO:0060590 ATPase regulator activity 5 3 0.16 0.00030

5 GO:0051087 chaperone binding 12 4 0.38 0.00041

6 GO:0015078 proton transmembrane transporter activity 74 16 2.36 0.00045

7 GO:0008233 peptidase activity 625 30 19.89 0.00054

8 GO:0004129 cytochrome-c oxidase activity 14 4 0.45 0.00078

9 GO:0030554 adenyl nucleotide binding 688 25 21.90 0.00100

10 GO:0004812 aminoacyl-tRNA ligase activity 43 6 1.37 0.00223

11 GO:0003756 protein disulfide isomerase activity 10 3 0.32 0.00324

12 GO:0022857 transmembrane transporter activity 500 37 15.92 0.00437

13 GO:0016780 phosphotransferase activity 12 3 0.38 0.00567

14 GO:0070006 metalloaminopeptidase activity 26 4 0.83 0.00863

15 GO:0097200 cysteine-type endopeptidase activity 14 3 0.45 0.00894

16 GO:0004586 ornithine decarboxylase activity 5 2 0.16 0.00947

17 GO:0004185 serine-type carboxypeptidase activity 15 3 0.48 0.01092

18 GO:0042277 peptide binding 50 5 1.59 0.01121

19 GO:0016679 oxidoreductase activity 6 2 0.19 0.01391

20 GO:0008484 sulfuric ester hydrolase activity 7 2 0.22 0.01907

21 GO:0008312 7S RNA binding 7 2 0.22 0.01907

22 GO:0005484 SNAP receptor activity 19 3 0.60 0.02118

23 GO:0008641 ubiquitin-like modifier activating enzyme 8 2 0.25 0.02490

24 GO:0016671 oxidoreductase activity 9 2 0.29 0.03135

25 GO:0010181 FMN binding 9 2 0.29 0.03135

26 GO:0003796 lysozyme activity 9 2 0.29 0.03135

27 GO:0046933 proton-transporting ATP synthase activity 9 2 0.29 0.03135

28 GO:0009055 electron transfer activity 54 8 1.72 0.03581

29 GO:0008137 NADH dehydrogenase (ubiquinone) activity 24 3 0.76 0.03940

**Gene Enrichment Analysis on list of genes that were significantly upregulated in sugar fed control injected virgin female midguts compared to sugar fed 20E injected virgin female midguts**

**Biological Process**

GO.ID Term Annotated Significant Expected weight01

1 GO:0006468 protein phosphorylation 263 44 15.86 2.4e-07

2 GO:0006355 regulation of transcription, DNA-template 532 64 32.09 8.6e-06

3 GO:0007169 transmembrane receptor protein tyrosine 32 9 1.93 0.00013

4 GO:0071526 semaphorin-plexin signaling pathway 6 4 0.36 0.00018

5 GO:0048841 regulation of axon extension 6 4 0.36 0.00018

6 GO:0006470 protein dephosphorylation 50 11 3.02 0.00026

7 GO:0051056 regulation of small GTPase mediated signal 42 12 2.53 0.00037

8 GO:0030010 establishment of cell polarity 7 4 0.42 0.00039

9 GO:0043547 positive regulation of GTPase activity 30 7 1.81 0.00043

10 GO:0006397 mRNA processing 111 14 6.69 0.00050

11 GO:0006606 protein import into nucleus 31 11 1.87 0.00082

12 GO:0018105 peptidyl-serine phosphorylation 20 6 1.21 0.00087

13 GO:0006607 NLS-bearing protein import into nucleus 9 4 0.54 0.00129

14 GO:0045596 negative regulation of cell differentiation 5 3 0.30 0.00199

15 GO:0000226 microtubule cytoskeleton organization 59 9 3.56 0.00297

16 GO:0000060 protein import into nucleus, translocation 6 3 0.36 0.00380

17 GO:0007179 transforming growth factor beta receptor 6 3 0.36 0.00380

18 GO:0032012 regulation of ARF protein signal transduction 6 3 0.36 0.00380

19 GO:1902532 negative regulation of intracellular signal 12 4 0.72 0.00437

20 GO:0035556 intracellular signal transduction 281 44 16.95 0.00450

21 GO:0006357 regulation of transcription by RNA polymerase 172 19 10.37 0.00498

22 GO:0006171 cAMP biosynthetic process 7 3 0.42 0.00635

23 GO:0007165 signal transduction 688 86 41.50 0.00652

24 GO:0051726 regulation of cell cycle 63 11 3.80 0.00936

25 GO:0032006 regulation of TOR signaling 8 3 0.48 0.00971

26 GO:0008360 regulation of cell shape 8 3 0.48 0.00971

27 GO:0017148 negative regulation of translation 15 4 0.90 0.01044

28 GO:0007010 cytoskeleton organization 120 19 7.24 0.01296

29 GO:0051649 establishment of localization in cell 279 27 16.83 0.01309

30 GO:0007094 mitotic spindle assembly checkpoint 9 3 0.54 0.01391

31 GO:0070588 calcium ion transmembrane transport 17 4 1.03 0.01655

32 GO:0048585 negative regulation of response to stimuli 21 7 1.27 0.01965

33 GO:0031175 neuron projection development 27 7 1.63 0.01969

34 GO:0030036 actin cytoskeleton organization 54 8 3.26 0.01995

35 GO:0045892 negative regulation of transcription 50 6 3.02 0.02486

36 GO:0016579 protein deubiquitination 30 5 1.81 0.03179

37 GO:0030334 regulation of cell migration 10 4 0.60 0.03185

38 GO:0051129 negative regulation of cellular component 29 5 1.75 0.03187

39 GO:0046471 phosphatidylglycerol metabolic process 5 2 0.30 0.03212

40 GO:0040013 negative regulation of locomotion 5 2 0.30 0.03212

41 GO:0010959 regulation of metal ion transport 5 2 0.30 0.03212

42 GO:0048015 phosphatidylinositol-mediated signaling 5 2 0.30 0.03212

43 GO:0048640 negative regulation of developmental growth 5 2 0.30 0.03212

44 GO:0000055 ribosomal large subunit export from nucleus 5 2 0.30 0.03212

45 GO:0051271 negative regulation of cellular component 5 2 0.30 0.03212

46 GO:0030335 positive regulation of cell migration 5 2 0.30 0.03212

47 GO:0051961 negative regulation of nervous system 5 2 0.30 0.03212

48 GO:0008299 isoprenoid biosynthetic process 13 3 0.78 0.03960

49 GO:0051656 establishment of organelle localization 28 5 1.69 0.04590

50 GO:0034968 histone lysine methylation 13 3 0.78 0.04615

51 GO:1901409 positive regulation of phosphorylation 6 2 0.36 0.04629

52 GO:0007064 mitotic sister chromatid cohesion 6 2 0.36 0.04629

53 GO:0046500 S-adenosylmethionine metabolic process 6 2 0.36 0.04629

54 GO:0044260 cellular macromolecule metabolic process 1834 164 110.62 0.04807

**Cellular Compartment**

GO.ID Term Annotated Significant Expected weight01

1 GO:0005856 cytoskeleton 192 31 11.33 1.1e-06

2 GO:0005622 intracellular 3281 250 193.62 9.8e-06

3 GO:0005634 nucleus 1327 111 78.31 0.00030

4 GO:0005737 cytoplasm 1724 111 101.74 0.00037

5 GO:0016459 myosin complex 18 6 1.06 0.00041

6 GO:0005643 nuclear pore 30 7 1.77 0.00146

7 GO:0030864 cortical actin cytoskeleton 5 3 0.30 0.00186

8 GO:0034399 nuclear periphery 5 3 0.30 0.00186

9 GO:0071004 U2-type prespliceosome 8 3 0.47 0.00913

10 GO:0031965 nuclear membrane 9 3 0.53 0.01311

11 GO:0005667 transcription factor complex 55 6 3.25 0.01650

12 GO:0043235 receptor complex 26 3 1.53 0.01928

13 GO:0005813 centrosome 28 5 1.65 0.02222

14 GO:0000407 phagophore assembly site 12 3 0.71 0.03009

15 GO:1902554 serine/threonine protein kinase complex 13 4 0.77 0.03058

16 GO:0000243 commitment complex 6 2 0.35 0.04446

17 GO:0008287 protein serine/threonine phosphatase com... 6 2 0.35 0.04446

18 GO:0000151 ubiquitin ligase complex 57 6 3.36 0.04963

**Molecular Function**

GO.ID Term Annotated Significant Expected weight01

1 GO:0005515 protein binding 2354 242 155.05 1.7e-13

2 GO:0005488 binding 5546 459 365.30 1.9e-07

3 GO:0004674 protein serine/threonine kinase activity 118 26 7.77 5.6e-06

4 GO:0005096 GTPase activator activity 58 14 3.82 1.7e-05

5 GO:0008536 Ran GTPase binding 16 7 1.05 3.5e-05

6 GO:0003700 DNA binding transcription factor activity 203 28 13.37 5.4e-05

7 GO:0008139 nuclear localization sequence binding 13 6 0.86 9.1e-05

8 GO:0004725 protein tyrosine phosphatase activity 37 10 2.44 9.7e-05

9 GO:0005524 ATP binding 684 69 45.05 0.00018

10 GO:0019901 protein kinase binding 12 5 0.79 0.00065

11 GO:0008565 protein transporter activity 32 8 2.11 0.00086

12 GO:0000166 nucleotide binding 1141 111 75.15 0.00112

13 GO:0003774 motor activity 57 9 3.75 0.00138

14 GO:0036002 pre-mRNA binding 6 3 0.40 0.00489

15 GO:0004016 adenylate cyclase activity 7 3 0.46 0.00814

16 GO:0004702 signal transducer, downstream of receptor 26 7 1.71 0.01011

17 GO:0004842 ubiquitin-protein transferase activity 93 14 6.13 0.01046

18 GO:0005086 ARF guanyl-nucleotide exchange factor activity 8 3 0.53 0.01240

19 GO:0004672 protein kinase activity 235 42 15.48 0.01904

20 GO:0008270 zinc ion binding 637 55 41.96 0.02133

21 GO:0098772 molecular function regulator 270 40 17.78 0.02201

22 GO:0051015 actin filament binding 17 4 1.12 0.02226

23 GO:0004114 3',5'-cyclic-nucleotide phosphodiesterase 10 3 0.66 0.02406

24 GO:0017137 Rab GTPase binding 31 6 2.04 0.02513

25 GO:0036459 thiol-dependent ubiquitinyl hydrolase activity 30 5 1.98 0.02714

26 GO:0003779 actin binding 54 10 3.56 0.03150

27 GO:0018024 histone-lysine N-methyltransferase activity 11 3 0.72 0.03150

28 GO:0016887 ATPase activity 209 17 13.77 0.03645

29 GO:0005085 guanyl-nucleotide exchange factor activity 58 12 3.82 0.03754

30 GO:0004707 MAP kinase activity 5 2 0.33 0.03790

31 GO:0042813 Wnt-activated receptor activity 5 2 0.33 0.03790

32 GO:0005158 insulin receptor binding 5 2 0.33 0.03790

33 GO:0008026 ATP-dependent helicase activity 56 4 3.69 0.03808

**Gene Enrichment Analysis of common 53 genes that are significantly upregulated upon mating and 20E injection in sugar fed females.**

**Biological Process**

GO.ID Term Annotated Significant Expected weight01

1 GO:0043171 peptide catabolic process 29 4 0.18 2.4e-05

2 GO:0006418 tRNA aminoacylation for protein translation 44 3 0.27 0.0023

3 GO:1901570 fatty acid derivative biosynthetic process 17 2 0.10 0.0059

4 GO:0009058 biosynthetic process 1474 14 8.91 0.0077

5 GO:0003333 amino acid transmembrane transport 23 2 0.14 0.0083

6 GO:0033387 putrescine biosynthetic process from ornithine 5 1 0.03 0.0299

7 GO:0042454 ribonucleoside catabolic process 5 1 0.03 0.0299

8 GO:0006767 water-soluble vitamin metabolic process 7 1 0.04 0.0416

9 GO:0006783 heme biosynthetic process 7 1 0.04 0.0416

10 GO:0061077 chaperone-mediated protein folding 8 1 0.05 0.0474

**Cellular Compartment**

GO.ID Term Annotated Significant Expected weight01

1 GO:0005791 rough endoplasmic reticulum 5 2 0.03 0.0003

2 GO:0005737 cytoplasm 1724 18 9.68 0.0011

3 GO:0005789 endoplasmic reticulum membrane 97 3 0.54 0.0166

4 GO:0016020 membrane 2818 23 15.83 0.0274

5 GO:0005885 Arp2/3 protein complex 7 1 0.04 0.0387

**Molecular Function**

GO.ID Term Annotated Significant Expected weight01

1 GO:0070006 metalloaminopeptidase activity 26 3 0.11 0.00018

2 GO:0004812 aminoacyl-tRNA ligase activity 43 3 0.18 0.00080

3 GO:0042277 peptide binding 50 3 0.21 0.00124

4 GO:0003755 peptidyl-prolyl cis-trans isomerase activity 23 2 0.10 0.00429

5 GO:0015171 amino acid transmembrane transporter activity 31 2 0.13 0.00772

6 GO:0016641 oxidoreductase activity 5 1 0.02 0.02131

7 GO:0070573 metallodipeptidase activity 5 1 0.02 0.02131

8 GO:0004586 ornithine decarboxylase activity 5 1 0.02 0.02131

9 GO:0016801 hydrolase activity, acting on ether bond 6 1 0.03 0.02552

10 GO:0005528 FK506 binding 7 1 0.03 0.02971

11 GO:0016779 nucleotidyltransferase activity 64 2 0.28 0.03063

12 GO:0003796 lysozyme activity 9 1 0.04 0.03804

13 GO:0010181 FMN binding 9 1 0.04 0.03804

14 GO:0016671 oxidoreductase activity 9 1 0.04 0.03804

15 GO:0003756 protein disulfide isomerase activity 10 1 0.04 0.04218

16 GO:0019239 deaminase activity 11 1 0.05 0.04630

**Gene Enrichment Analysis of common 23 genes that are significantly downregulated upon mating and 20E injection in sugar fed females.**

**Biological Process**

GO.ID Term Annotated Significant Expected weight01

1 GO:0007586 digestion 10 2 0.03 0.00032

2 GO:0046471 phosphatidylglycerol metabolic process 5 1 0.01 0.01382

3 GO:0000055 ribosomal large subunit export from nucleus 5 1 0.01 0.01382

4 GO:0000479 endonucleolytic cleavage of tricistronic 8 1 0.02 0.02203

5 GO:0016075 rRNA catabolic process 11 1 0.03 0.03017

6 GO:0008654 phospholipid biosynthetic process 43 2 0.12 0.03366

7 GO:0008299 isoprenoid biosynthetic process 13 1 0.04 0.03556

8 GO:0042254 ribosome biogenesis 156 5 0.43 0.03847

9 GO:0000027 ribosomal large subunit assembly 15 1 0.04 0.04093

**Cellular Compartment**

GO.ID Term Annotated Significant Expected weight01

1 GO:0030688 preribosome, small subunit precursor 5 1 0.01 0.014

2 GO:0005730 nucleolus 87 2 0.25 0.026

3 GO:0000177 cytoplasmic exosome (RNase complex) 9 1 0.03 0.026

4 GO:0000176 nuclear exosome (RNase complex) 11 1 0.03 0.031

5 GO:0000407 phagophore assembly site 12 1 0.03 0.034

6 GO:1902554 serine/threonine protein kinase complex 14 1 0.04 0.040

**Molecular Function**

GO.ID Term Annotated Significant Expected weight01

1 GO:0000175 3'-5'-exoribonuclease activity 7 1 0.02 0.019

2 GO:0004659 prenyltransferase activity 8 1 0.02 0.021

3 GO:0019901 protein kinase binding 12 1 0.03 0.032

4 GO:0016780 phosphotransferase activity 12 1 0.03 0.032

5 GO:0004497 monooxygenase activity 110 2 0.30 0.035

6 GO:0030515 snoRNA binding 14 1 0.04 0.037

**Gene enrichment analysis on non-overlapped genes (only regulated upon mating) on sugar fed females midguts (p <0.05)**

**Biological Process**

GO.ID                        Term                         Annotated        Significant     Expected weight01

__________________________________________________________________________________________________________

GO:0009813              flavonoid biosynthetic process        19           8     0.78  3.8e-07

GO:0052696            flavonoid glucuronidation        19           8     0.78  3.8e-07

GO:0042742             defense response to bacterium        10           5     0.41  2.4e-05

GO:0009253             peptidoglycan catabolic process         9           4     0.37  0.00030

GO:0046323               glucose import 17           5     0.70  0.00047

GO:0055085               transmembrane transport     426          31    17.55  0.00063

GO:0032787       monocarboxylic acid metabolic process       108          16     4.45  0.00113

GO:0045087               innate immune response     31           6     1.28  0.00142

GO:0006749               glutathione metabolic process        37           6     1.52  0.00365

GO:0009070 serine family amino acid biosynthetic process        10           3     0.41  0.01547

GO:0009074 aromatic amino acid family catabolic process         5           2     0.21  0.01556

GO:0055114               oxidation-reduction process       517          30    21.30  0.01682

GO:0000027            ribosomal large subunit assembly        15           3     0.62  0.02176

GO:1901657         glycosyl compound metabolic process        49           5     2.02  0.02250

GO:0031126               snoRNA 3'-end processing         6           2     0.25  0.02271

GO:0006783               heme biosynthetic process         7           2     0.29 0.03094

GO:0000154               rRNA modification 17           3     0.70  0.03997

GO:0000463 maturation of LSU-rRNA from tricistronic transcript  8           2     0.33  0.04015

GO:0000479 endonucleolytic cleavage of tricistronic  transcript 8           2     0.33  0.04015

GO:0006414               translational elongation       20           3     0.82  0.04690

**Cellular Compartment**

GO.ID                        Term                         Annotated        Significant     Expected weight01

__________________________________________________________________________________________________________

GO:0043231    intracellular membrane-bounded organelle      2053          60    69.54  9.9e-05

GO:0005887       integral component of plasma membrane       271          20     9.18   0.0012

GO:0044452             nucleolar part        30           5     1.02   0.0048

GO:1902495           transmembrane transporter complex        46           3     1.56   0.0066

GO:0005732 small nucleolar ribonucleoprotein complex        12           3     0.41   0.0067

GO:0022625           cytosolic large ribosomal subunit        43           5     1.46   0.0143

**Molecular Function**

GO.ID                        Term                         Annotated        Significant     Expected weight01

__________________________________________________________________________________________________________

GO:0008745 N-acetylmuramoyl-L-alanine amidase activity         9           4     0.31  0.00016

GO:0005355 glucose transmembrane transporter activity        17           5     0.59  0.00022

GO:0005351      carbohydrate:proton symporter activity        20           5     0.70  0.00051

GO:0003824             catalytic activity      3379         156   118.25  0.00106

GO:0015020            glucuronosyltransferase activity        14           4     0.49  0.00111

GO:0008194            UDP-glycosyltransferase activity        41           9     1.43  0.00206

GO:0004364            glutathione transferase activity        27           5     0.94  0.00217

GO:0060089               molecular transducer activity       286           7    10.01  0.00370

GO:0004602             glutathione peroxidase activity        21           4     0.73  0.00549

GO:0003746      translation elongation factor activity        13           3     0.45  0.00934

GO:0022857          transmembrane transporter activity       498          29    17.43  0.01323

GO:0004190        aspartic-type endopeptidase activity         6           2     0.21  0.01668

GO:0008422              beta-glucosidase activity         6           2     0.21  0.01668

GO:0005215            transporter activity       621          40    21.73  0.01677

GO:0016411     acylglycerol O-acyltransferase activity         7           2     0.24  0.02281

GO:0071949      FAD binding         8           2     0.28  0.02972

GO:0016671 oxidoreductase activity, acting on a sulfur         9           2     0.31  0.03734

GO:0005548           phospholipid transporter activity         9           2     0.31  0.03734

GO:0043169              cation binding      1532          48    53.61  0.03951

GO:0016709 oxidoreductase activity, acting on paired donor        10           2     0.35  0.04562

**Gene enrichment analysis on non-overlapped genes (only regulated upon 20E/Control injection) on sugar fed females midguts (p <0.05)**

**Biological Process**

GO.ID                        Term                         Annotated        Significant     Expected weight01

__________________________________________________________________________________________________________

GO:0006397            mRNA processing       111          19    9.94  0.00039

GO:0000226       microtubule cytoskeleton organization        59          10     5.28  0.00039

GO:0006468             protein phosphorylation       263          45    23.56  0.00076

GO:0048841 regulation of axon extension         6           4     0.54  0.00082

GO:0071526         semaphorin-plexin signaling pathway         6           4     0.54  0.00082

GO:0006470             protein dephosphorylation        50          12     4.48  0.00161

GO:0006890 retrograde vesicle-mediated transport         11           5     0.99  0.00166

GO:0051056 regulation of small GTPase mediated signal        42          12     3.76  0.00173

GO:0030010              establishment of cell polarity         7           4     0.63  0.00179

GO:0007169 transmembrane receptor protein tyrosine kinase     32           9     2.87  0.00187

GO:0015991     ATP hydrolysis coupled proton transport        22           7     1.97  0.00230

GO:0043547      positive regulation of GTPase activity        30           7     2.69  0.00262

GO:0006511 ubiquitin-dependent protein catabolic process       117          18    10.48  0.00283

GO:0032006              regulation of TOR signaling         8           4     0.72  0.00332

GO:0007010           cytoskeleton organization       120          24    10.75  0.00519

GO:0006607     NLS-bearing protein import into nucleus         9           4     0.81  0.00556

GO:0045596 negative regulation of cell differentiation     5           3     0.45  0.00623

GO:0006606             protein import into nucleus        31          11     2.78  0.00631

GO:0018105             peptidyl-serine phosphorylation        20           6     1.79  0.00654

GO:0006357 regulation of transcription by RNA polymerase II   172          23    15.41  0.00703

GO:0045047           protein targeting to ER        12           5     1.07  0.00795

GO:0032956 regulation of actin cytoskeleton organization        27           5     2.42  0.00799

GO:0015986      ATP synthesis coupled proton transport        21           6     1.88  0.00847

GO:0006355 regulation of transcription, DNA-template       532          70    47.65  0.00988

GO:0051603 proteolysis involved in cellular protein catabolic    141          24    12.63  0.01010

GO:0051649       establishment of localization in cell       278          44    24.90  0.01063

GO:0000060 protein import into nucleus, translocation         6           3     0.54  0.01163

GO:0032012 regulation of ARF protein signal transduction         6           3     0.54  0.01163

GO:0007179 transforming growth factor beta receptor          6           3     0.54  0.01163

GO:1902532 negative regulation of intracellular signal     12           4     1.07  0.01759

GO:0006171             cAMP biosynthetic process         7           3     0.63  0.01902

GO:0006206     pyrimidine nucleobase metabolic process         7           3     0.63  0.01902

GO:0006465              signal peptide processing         7           3     0.63  0.01902

GO:0006730           one-carbon metabolic process        13           4     1.16  0.02365

GO:0035556           intracellular signal transduction       281          50    25.17  0.02584

GO:0051726        regulation of cell cycle        63          13     5.64  0.02773

GO:0007015          actin filament organization        34           6     3.05  0.02834

GO:0032984      protein-containing complex disassembly        28           5     2.51  0.02840

GO:0006122 mitochondrial electron transport         8           3     0.72  0.02843

GO:0008360          regulation of cell shape         8           3     0.72  0.02843

GO:0016192          vesicle-mediated transport       194          26    17.38  0.03144

GO:0006812           cation transport       190          25    17.02  0.03790

GO:0009966           regulation of signal transduction       105          24     9.41  0.03823

GO:0015718               monocarboxylic acid transport         9           3     0.81  0.03986

GO:0007094         mitotic spindle assembly checkpoint         9           3     0.81  0.03986

GO:1902600              proton transmembrane transport        40          13     3.58  0.04140

GO:0048585 negative regulation of response to stimuli        21           7     1.88  0.04202

GO:0031175               neuron projection development        27           6     2.42  0.04225

GO:0007088      regulation of mitotic nuclear division        17           5     1.52  0.04226

GO:0016579         protein deubiquitination        30           6     2.69  0.04671

GO:0006891      intra-Golgi vesicle-mediated transport        16           4     1.43  0.04861

**Cellular Compartment**

GO.ID                        Term                         Annotated        Significant     Expected weight01

__________________________________________________________________________________________________________

GO:0005737              cytoplasm      1724         219   166.09    2e-06

GO:0005856   cytoskeleton       192          40    18.50    2e-06

GO:0033179 proton-transporting V-type ATPase, V0 domain        9           5     0.87  0.00074

GO:0019773 proteasome core complex, alpha-subunit complex   7 4     0.67  0.00235

GO:0005622           intracellular      3281         386   316.09  0.00498

GO:0016459       myosin complex        18           6     1.73  0.00526

GO:0034399         nuclear periphery         5           3     0.48  0.00767

GO:0030864            cortical actin cytoskeleton         5           3     0.48  0.00767

GO:0030126           COPI vesicle coat         6           3     0.58  0.01424

GO:0016471 vacuolar proton-transporting V-type ATPase          6           3     0.58  0.01424

GO:0000932        P-body        17           5     1.64  0.01893

GO:0005643          nuclear pore        30           7     2.89  0.02119

GO:0005885     Arp2/3 protein complex         7           3     0.67  0.02316

GO:0000276 mitochondrial proton-transporting ATP synthase     7           3     0.67  0.02316

GO:0000151      ubiquitin ligase complex        57           8     5.49  0.03044

GO:1905368        peptidase complex        45          11     4.34  0.03374

GO:0005839      proteasome core complex        15           7     1.45  0.03392

GO:0071004     U2-type prespliceosome         8           3     0.77  0.03445

GO:0031965   nuclear membrane         9           3     0.87  0.04806

GO:0030117           membrane coat        28           8     2.70  0.04814

GO:0043235          receptor complex        26           3     2.50  0.04892

**Molecular Function**

GO.ID                        Term                         Annotated        Significant     Expected weight01

__________________________________________________________________________________________________________

GO:0005488            binding      5546         564   501.21  3.2e-09

GO:0005515        protein binding      2354         282   212.74  2.5e-07

GO:0004298       threonine-type endopeptidase activity        15           7     1.36  0.00016

GO:0008536        Ran GTPase binding        16           7     1.45  0.00026

GO:0004725      protein tyrosine phosphatase activity        37          11     3.34  0.00029

GO:0005524         ATP binding       684          87    61.82  0.00049

GO:0005096           GTPase activator activity        58          14     5.24  0.00050

GO:0008139       nuclear localization sequence binding        13           6     1.17  0.00053

GO:0004674    protein serine/threonine kinase activity       105          22     9.49  0.00069

GO:0008565           protein transporter activity        32           9     2.89  0.00160

GO:0003700 DNA-binding transcription factor activity       203          30    18.35  0.00188

GO:0046961 proton-transporting ATPase activity, rotational        16           6     1.45  0.00194

GO:0003723         RNA binding       332          42    30.00  0.00567

GO:0060590       ATPase regulator activity         5           3     0.45  0.00640

GO:0051117           ATPase binding         5           3     0.45  0.00640

GO:0003774        motor activity        57           9     5.15  0.00687

GO:0030554          adenyl nucleotide binding       701          90    63.35  0.00756

GO:0000166           nucleotide binding      1146         136   103.57  0.00972

GO:0036002             pre-mRNA binding         6           3     0.54  0.01194

GO:0008233       peptidase activity       625          36    56.48  0.01309

GO:0016874          ligase activity       111          14    10.03  0.01743

GO:0051087         chaperone binding        12           4     1.08  0.01816

GO:0019901            protein kinase binding        12           4     1.08  0.01816

GO:0005543         phospholipid binding        51           8     4.61  0.01943

GO:0004016      adenylate cyclase activity         7           3     0.63  0.01950

GO:0008484           sulfuric ester hydrolase activity         7           3     0.63  0.01950

GO:0004842      ubiquitin-protein transferase activity        93          17     8.40  0.02224

GO:0004672      protein kinase activity       235          42    21.24  0.02696

GO:0005086 ARF guanyl-nucleotide exchange factor activity       8           3     0.72  0.02913

GO:0004129               cytochrome-c oxidase activity        14           4     1.27  0.03177

GO:0098772           molecular function regulator       270          45    24.40  0.04112

GO:0022857          transmembrane transporter activity       498          49    45.01  0.04488
